# Unveiling the Anticancer Potential of Urolithin A in Colorectal Cancer: A Systematic Review

**DOI:** 10.32604/or.2025.070276

**Published:** 2026-01-19

**Authors:** Mariana Francisco, Fernando Mendes, Diana Martins, Joana Liberal

**Affiliations:** 1 Polytechnic University of Coimbra, ESTESC, UCPCBL, Rua 5 de Outubro, SM Bispo, Apartado, Coimbra, 3046-854, Portugal; 2 H&TRC—Health & Technology Research Center, Coimbra Health School, Polytechnic University of Coimbra, Coimbra, 3045-043, Portugal; 3 Coimbra Institute for Clinical and Biomedical Research (iCBR) Area of Environment Genetics and Oncobiology (CIMAGO), Biophysics Institute of Faculty of Medicine, University of Coimbra, Coimbra, 3045-043, Portugal; 4 Center for Innovative Biomedicine and Biotechnology (CIBB), University of Coimbra, Coimbra, 3045-043, Portugal; 5 European Association of Biomedical Scientists, Brussels, 1000, Belgium

**Keywords:** Colorectal cancer, urolithin A, 3,8-dihydroxy-6H-dibenzo(b,d)pyran-6-one, anticancer effects, systematic review

## Abstract

**Objectives:**

Colorectal cancer (CRC) is a major global health burden, and Urolithin A (Uro-A) has emerged as a promising anticancer agent. This systematic review aims to synthesize current *in vitro* evidence on the anticancer effects of Uro-A in CRC, highlighting effective concentration ranges, exposure times, relevant outcomes, and underlying molecular mechanisms.

**Methods:**

Following PRISMA 2020 guidelines, a systematic search was conducted in PubMed, Scopus, and Web of Science using the following strategy: (colorectal cancer) AND (urolithin a) OR (3,8-dihydroxy-6H-dibenzo(b,d)pyran-6-one). Eligibility criteria were defined by the PICO framework: (P) *in vitro* CRC cell models; (I) Uro-A alone or combined treatments; (C) No intervention, vehicle or other treatments; (O) Relevant anticancer outcomes of Uro-A in CRC. Only original, full-text, *in vitro* studies in English were included. Risk of bias was assessed using ToxRTool. A qualitative synthesis was performed due to the heterogeneity of the included studies.

**Results:**

Fifteen studies met inclusion criteria, involving CRC cell lines (Caco-2, HCT-116, HT-29, SW480, SW620) and normal colon fibroblasts (CCD18-Co). Uro-A inhibited CRC cell proliferation, clonogenic growth, cancer stem cells properties, migration, and invasion, and induced cell cycle arrest, apoptosis, autophagy, and senescence, through modulation of key signaling pathways and proteins. Co-treatments with conventional chemotherapeutics and microbiota-derived metabolites showed additive or synergistic effects.

**Discussion:**

The findings support Uro-A’s potential as a preventive or adjuvant agent in CRC treatment. However, preclinical nature of the evidence and methodological heterogeneity hinder clinical extrapolation to *in vivo* contexts. Human clinical trials are necessary to overcome these limitations.

**Other:**

This review was registered in PROSPERO (CRD420251070874) and supported by FCT/MCTES UIDP/05608/2020 and UIDB/05608/2020. Institutional.

## 1 Introduction

Among all types of malignancies, colorectal cancer (CRC) remains one of the most prevalent and lethal. In 2022, more than 1.92 million new cases and nearly 904,000 deaths were reported in both males and females, ranking CRC the third most common type of cancer and the second leading cause of cancer-related deaths worldwide [1].

Geographically, the incidence rate was highest in Europe, Australia/New Zealand and North America, respectively. This can be partially explained by the higher prevalence of risk factors in these regions, such as genetic predisposition, older age, sedentary behavior, alcohol consumption, smoking, high intake of processed foods and fats, as well as low consumption of fruits and vegetables [1–3].

Although conventional treatments for CRC, such as chemotherapy, molecular-targeted therapy, immunotherapy, radiotherapy, and surgery, have improved patient outcomes, they still present relevant limitations [4]. Notably, significant side effects and frequent development of drug resistance continue to be major challenges [4], which underscored the need to identify therapeutic alternatives capable of enhancing the efficacy while minimizing the adverse impacts of current approaches. In this context, nutraceuticals and dietary strategies have emerged as promising candidates, as they may exert a dual role-preventive benefits and therapeutic effects—depending on their concentration and specific clinical context [5,6].

In fact, several studies have highlighted the importance of implementing healthy eating habits, given that diet, a modifiable risk factor, plays an active role in the prevention of CRC [5]. Among the various dietary patterns, the Mediterranean diet has gained particular interest due to its high source of nutraceuticals—bioactive compounds which, beyond their nutritional value, also exhibit preventive and therapeutic properties, especially in the context of cardiovascular diseases, immune system disorders, and cancer [5,6]. Nutraceuticals can be classified based on their nature, origin, and application. Among them, phytochemicals stand out as plants’ secondary metabolites, including phenols, terpenoids and alkaloids, which contribute to their defense and protection against environmental stress [6].

Ellagitannins (ET) are polyphenolic organic compounds widely present in various edible plants such as walnuts, pomegranates and berries, and beverages like herbal teas and wine [7], and have drawn significant attention from the scientific community due to their anticancer, anti-inflammatory, and antioxidant properties [8]. Since ETs are hydrolysable tannins, they have the ability to undergo hydrolysis under enzymatic or acidic conditions [8,9] and produce the intermediate compound hexahydroxydiphenoyl (HHDP), which is subsequently converted into ellagic acid (EA) [7]. However, the chemical structure of ET and EA—particularly their polarity and high molecular weight—gives them low bioavailability [10,11]. In this regard, after the intake of ET-rich products, EA is metabolized in the gut by microbiota, leading to 6H-dibenzo[b,d]pyran-6-one derivatives, commonly known as urolithins (Uro) [10]. In fact, as these compounds are more easily absorbed due to their higher lipophilicity, they are the ones responsible for the health benefits attributed to their precursors [11].

**Figure 1:**
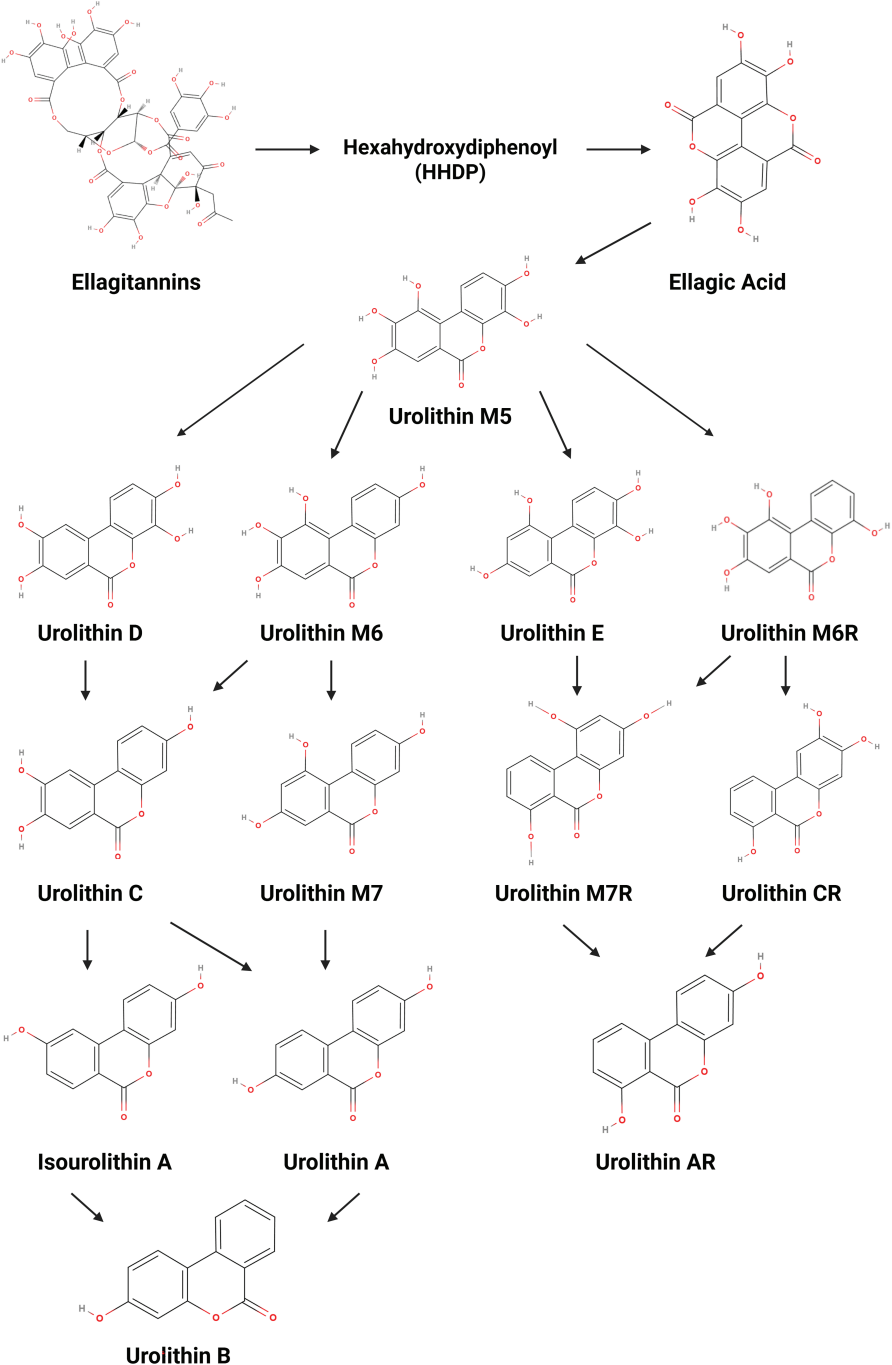
Metabolic conversion of ellagitannins and ellagic acid into urolithins. Created with BioRender.com

It is well established that the type of Uro produced, as well as its concentration and biological activity, is influenced by the composition of the host’s gut microbiota [12,13]. Considering this interindividual variability, individuals can be stratified according to three distinct metabolic phenotypes: metabotype A produces Uro-A and its conjugates; metabotype B produces Uro-A, isourolithin A (IsoUro-A) and Uro-B, and metabotype 0 does not produce any Uro [10,13]. The synthesis pathway of Uro consists of multiple decarboxylation and dihydroxylation reactions, beginning with the production of Uro-M5 (pentahydroxy-Uro), followed by Uro-D, Uro-E, Uro-M6 or Uro-M6R (tetrahydroxy-Uro), Uro-C, Uro-CR, Uro-M7 or Uro-M7R (trihydroxy-Uro), Uro-A, IsoUro-A or Uro-AR (dihydroxy-Uro), and concluding with Uro-B (monohydroxy-Uro) (Fig. 1) [7,8].

According to several reports, Uros have already been identified in different human tissues, including the prostate, colon and breast, as well as in fluids such as feces, blood, urine and breastmilk [7,8]. Furthermore, Uro-A and Uro-B are the ones mostly found in the gut, reaching concentrations in the micromolar range in both normal and malignant tissues, with Uro-A exhibiting the highest biological activity [8,13]. In this context, Uro-A has been described as a promising anticancer agent in the treatment and prevention of CRC.

In a dose- and time-dependent manner, Uro-A has been reported to inhibit cell proliferation through cell cycle arrest and activation of apoptosis pathways, induce cellular senescence and autophagy, as well as suppress cell migration and invasion by modulating key proteins and critical signaling pathways in carcinogenesis [13–15]. Although several *in vitro* studies have described the anticancer properties of Uro-A in CRC, data on its effective concentrations, exposure times, and molecular targets remain fragmented.

In light of these crucial findings, this systematic review aims to synthesize the current scientific evidence of Uro-A in CRC by identifying the therapeutic concentration ranges and exposure periods employed in *in vitro* models of CRC, characterizing outcomes with potential impact on carcinogenesis, and elucidating the molecular mechanisms underlying the anticancer activity of Uro-A. By addressing these knowledge gaps, this review aims to provide a robust scientific foundation for future studies and clinical trials, ultimately contributing to the development of new preventive and therapeutic strategies for CRC.

## 2 Materials and Methods

This systematic review followed the guidelines outlined by the Preferred Reporting Items for Systematic Reviews and Meta-Analyses (PRISMA) and was reported in accordance with both PRISMA 2020 checklists (Supplementary Material) [16]. The protocol for this work was registered in the International Prospective Register of Systematic Reviews (PROSPERO; registration number CRD420251070874) [17].

### 2.1 Information Sources and Search Strategy

The literature search was conducted in three electronic databases, including PubMed, Scopus and Web of Science in April 2025. The search strategy was performed using the following combination of keywords, Medical Subject Headings (MeSH) and Boolean operators: (colorectal cancer) AND ((urolithin a) OR (3,8-dihydroxy-6H-dibenzo(b,d)pyran-6-one)). The search period included only articles published between April 2014 and June 2024 (Table 1).

**Table 1:** Search strategies for each database, including filters and limits used

Database	Search query
PubMed	((“colorectal neoplasms”[MeSH Terms] OR “colorectal cancer”[All Fields] OR (“colorectal”[All Fields] AND (“neoplasms”[All Fields] OR “cancer”[All Fields]))) AND (“urolithin”[All Fields] OR “urolithins”[All Fields] OR “3 8 dihydroxy 6h dibenzo b d pyran 6 one”[All Fields]) AND 2014/01/01:2025/12/31[Date-Publication] AND “loattrfull text”[Filter]) AND ((fft[Filter]) AND (2014:2024[pdat]))
Scopus	(TITLE-ABS-KEY (“colorectal cancer”) AND TITLE-ABS-KEY (“urolithin A”)) AND PUBYEAR > 2013 AND PUBYEAR < 2025 AND (LIMIT-TO (LANGUAGE, “English”))
Web of Science	TS = (“colorectal cancer”) AND TS = (“urolithin a”) OR TS = (“3,8-dihydroxy-6H-dibenzo(b,d)pyran-6-one”) and English (Languages) and 2014 or 2015 or 2016 or 2017 or 2018 or 2019 or 2020 or 2021 or 2022 or 2023 or 2024 (Publication Years)

### 2.2 Eligibility Criteria

The inclusion criteria were defined according to the following Patient/Population, Intervention, Comparison, and Outcome (PICO) framework: (P) *In vitro* models of CRC, including established cell lines; (I) Individual or combined use of Uro-A as a preventive or therapeutic agent; (C) No intervention, vehicle or other treatments; (O) Evaluation of the anticancer potential of Uro-A in the prevention and treatment of CRC, including its effects on carcinogenesis, tumor progression, cell proliferation, apoptosis, autophagy, senescence, and other relevant cellular or molecular endpoints.

Studies that met the following criteria were included: (1) Full-text articles; (2) Original experimental studies conducted *in vitro* in human cells; (3) Studies published in English. The exclusion criteria were: (1) Literature reviews; (2) Meta-analyses; (3) Narrative reviews; (4) Studies with human participants *in vivo* or *ex vivo*; (5) Studies with animal models *in vivo* or *ex vivo;* (6) *In silico* studies.

### 2.3 Study Selection

The retrieved articles were imported into the Mendeley Reference Management Software (version 1.19.8) to remove duplicates. The articles were screened independently by two reviewers using the PICO Portal platform, following a hierarchical approach in which titles, abstracts, and full texts were reviewed sequentially according to the eligibility criteria. Any disagreements were solved through discussion between both authors or consultation with a third reviewer.

### 2.4 Data Extraction

The following data were independently and manually extracted by two reviewers from each selected study using an Excel spreadsheet. Discrepancies were resolved by discussion or consultation with a third reviewer. Extracted data included: (1) Name of the author; (2) Publication year; (3) Country; (4) Study type; (5) Experimental model; (6) Type of intervention; (7) Control or comparison group; (8) Concentration of compounds administered; (9) Duration of the treatment; (10) Analytical methods used to assess the outcome; (11) Outcomes.

### 2.5 Bias Assessment

The risk of bias was assessed independently by two reviewers using the Toxicological data Reliability Assessment Tool (ToxRTool) [18] for *in vitro* data to determine the methodological quality of the selected studies. Any discrepancies were solved by discussion or consultation with a third researcher. This software encompasses a total of eighteen criteria for *in vitro* studies, each of which can be scored as either “0” (criterion not met) or “1” (criterion met). The criteria are organized into five categories: (I) Test substance identification; (II) Test system characterization; (III) Study design description; (IV) Study results documentation; and (V) Plausibility of study design and results. Data reliability is determined according to the Klimisch category, based on the total score of the evaluated criteria. A total score of 15–18 corresponds to category 1 (reliable without restrictions), 11–14 to category 2 (reliable with restrictions), and scores below 11 to category 3 (not reliable). In addition, the tool identifies certain minimum requirements, highlighted in red, that are considered essential for the study’s reliability. Detailed information on the risk of bias assessment is provided in Supplementary Table S1.

### 2.6 Synthesis Methods

A qualitative synthesis was performed due to the heterogeneity of the included studies. Key findings from each study were summarized to report the measured outcomes, highlight differences in results between studies, and identify gaps in the current evidence.

## 3 Results

### 3.1 Search Results

**Figure 2:**
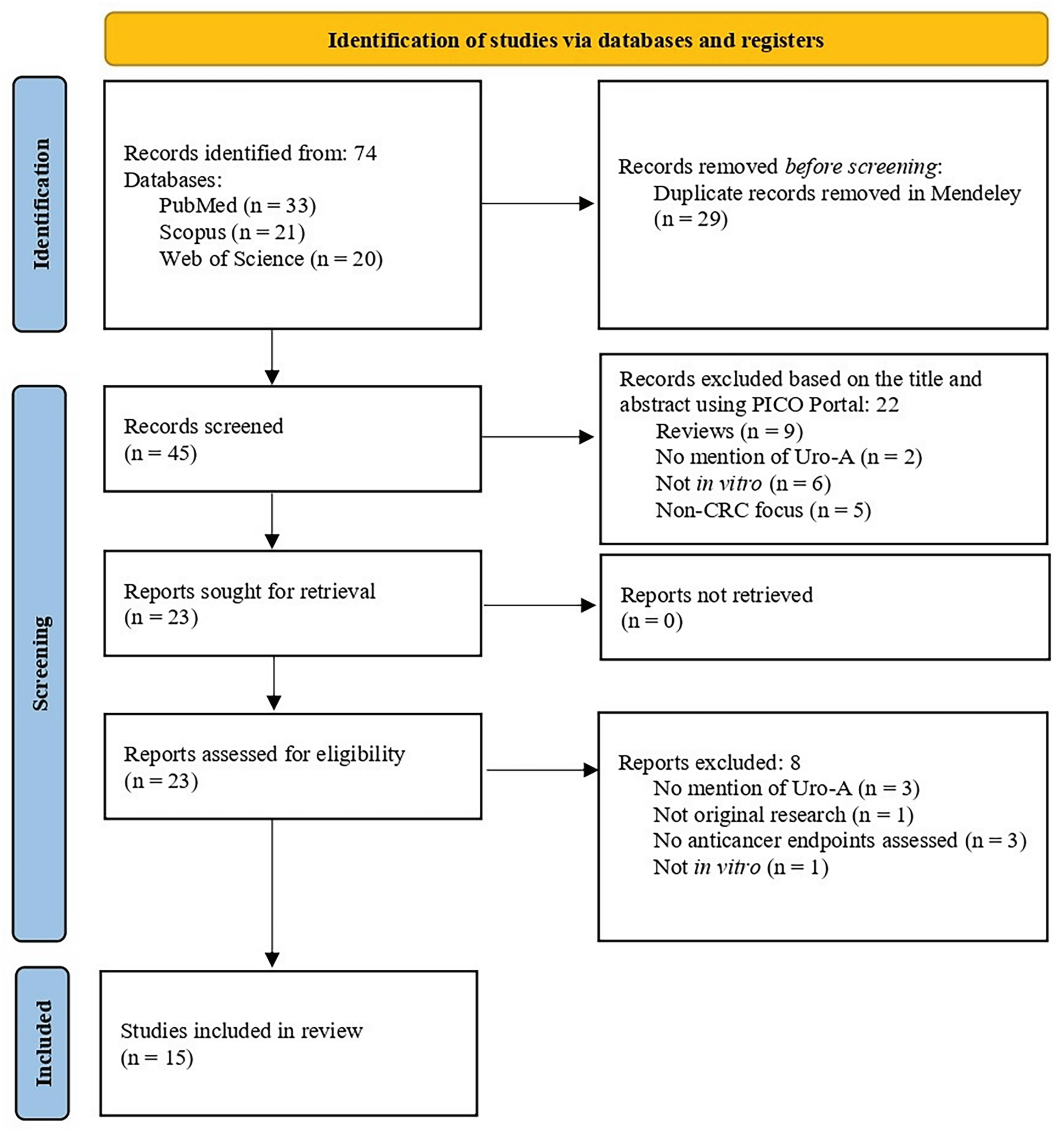
PRISMA 2020 flow diagram for systematic reviews

The search strategy yielded a total of seventy-four articles across the three databases. After removing duplicates (n = 29) and screening the articles based on titles and abstracts, using the Mendeley and PICO Portal platforms, respectively, twenty-three articles remained. A total of eight articles were excluded after full text screening. Therefore, fifteen articles were included in this systematic review (Fig. 2).

### 3.2 Study Characteristics

The included studies were performed using *in vitro* models. Eight studies tested Uro-A alone, while seven studies evaluated its effects in combination with chemotherapeutics, including 5-Fluorouracil (5-FU) (n = 2), 5^′^-Deoxy-5-fluorouridine (5^′^-DFUR) (n = 1) and oxaliplatin (n = 1), mixtures of ET metabolites mimicking metabotypes A (MPhA) and B (MPhB) (n = 3), and sodium butyrate (SB) (n = 1), a short-chain fatty acid produced by the gut microbiota. All studies were performed on human CRC cell lines, namely Caco-2 (American Type Culture Collection (ATCC®) HTB-37™), HCT-116 (ATCC® CCL-247™), HT-29 (ATCC® HTB-38™), SW480 (ATCC® CCL-228™), and SW620 (ATCC® CCL-227™) [19]. Caco-2, HT-29, and SW480 are colorectal adenocarcinoma cell lines, HCT-116 is a colorectal carcinoma cell line, and SW620 is a metastatic cell line derived from a lymph node of the same patient from whom SW480 was originally established. CCD18-Co (ATCC® CRL-1459™) normal colon fibroblasts were also included in a subset of experiments for comparative analysis [19]. The characteristics of the included studies are presented in Table 2.

**Table 2:** Characteristics of the included studies (n = 15): author, year, experimental model, intervention, assay type and outcomes

Author, Year	Experimental model (Human cell lines)	Intervention	Assay type	Outcome
González-Sarrías et al. [20], 2014	Caco-2, HT-29 and SW480	Uro-A	MTT; Trypan blue exclusion; Flow cytometry	Antiproliferative activity; cell cycle arrest
Cho et al. [21], 2015	HT-29	Uro-A	MTT; Western blot; Annexin V-FITC/PI; JC-1; Colorimetric caspase protease assay	Antiproliferative activity; cell cycle arrest; apoptosis
González-Sarrías et al. [22], 2015	Caco-2, HT-29 and SW480	Uro-A and 5-FU or 5^′^-DFUR	MTT; Flow cytometry; Western blot; Annexin V/PI; Hoechst 33242	Antiproliferative activity; cell cycle arrest; apoptosis
González-Sarrías et al. [23], 2016	Caco-2, HT-29, SW480 and CCD18-Co	Uro-A; MPhA, and MPhB	Trypan blue exclusion; Flow cytometry; Annexin V/PI	Antiproliferative activity; cell cycle arrest; apoptosis
Núñez-Sánchez et al. [24], 2016	Caco-2	MPhA and MPhB	Colonosphere formation assay; Aldefluor; Flow cytometry	Suppression of CSC-related properties
González-Sarrías et al. [25], 2017	Caco-2 and CCD18-Co	Uro-A	MTT; Trypan blue exclusion; Flow cytometry; Annexin V/PI	Antiproliferative activity; cell cycle arrest; apoptosis
Zhao et al. [26], 2018	SW620	Uro-A	MTT; Flow cytometry; Annexin V-FITC/PI; Chamber migration assay; Colorimetric assay; Transmission electron microscopy; GFP-LC3; Western blot	Antiproliferative activity; cell cycle arrest; apoptosis; cell migration and invasion suppression; autophagy
Tortora et al. [27], 2018	HT-29 and HCT-116	Uro-A and SB	MTS; Western blot	Antiproliferative activity; apoptosis
Norden et al. [28], 2019	HCT-116	Uro-A and oxaliplatin	Crystal violet staining; Flow cytometry; X-gal; CPD	Antiproliferative activity; cell cycle arrest; cellular senescence
Giménez-Bastida et al. [13], 2020	Caco-2, HCT-116, HT-29 and CCD18-Co	Uro-A; MPhA and MPhB	Clonogenic assay; Flow cytometry; FDG; X-gal; Western blot	Anti-clonogenic growth; cell cycle arrest; cellular senescence
El-Wetidy et al. [29], 2021	HT-29, SW480, SW620 and CCD18-Co	Uro-A	MTT; PI; Flow cytometry; Western blot; Annexin V/PI	Antiproliferative activity; cell cycle arrest; apoptosis
Ghosh et al. [30], 2022	HCT-116, SW480, HCT-116-FUR, SW480-FUR	Uro-A and 5-FU	MTT; Annexin V-FITC/PI; Flow cytometry; Western blot; Scratch assay; Three-dimensional invasion assay	Antiproliferative activity; apoptosis; cell migration and invasion suppression
Lin et al. [12], 2023	HT-29, SW480, SW620 and HCT-116	Uro-A	MTT	Antiproliferative activity
El-Wetidy et al. [15], 2024	HT-29, SW480, and SW620	Uro-A	AlamarBlue; Wound healing assay; Western blot	Antiproliferative activity; anti-clonogenic growth; cell migration and invasion suppression
Tiwari et al. [31], 2024	HT-29	Uro-A	MTT	Antiproliferative activity

Note: 5^′^-DFUR, 5^′^-Deoxy-5-fluorouridine; 5-FU, 5-Fluorouracil; Annexin V/PI, annexin V/propidium iodide; Annexin V-FITC/PI, annexin V-fluorescein isothiocyanate/propidium iodide; CPD, cumulative population doublings; CSC, cancer stem cells; FDG, 5-dodecanoylaminofluorescein di-*β*-D-galactopyranoside; FUR, 5-Fluorouracil chemoresistant; GFP-LC3, green fluorescent protein-microtubule-associated protein 1 light chain 3; JC-1, 5,5^′^,6,6^′^-tetrachloro-1,1^′^,3,3^′^-tetraethyl-benzimidazolocarbocyanine iodide; MPhA, mixtures of ET metabolites mimicking metabotype A; MPhB, mixtures of ET metabolites mimicking metabotype B; MTS, 3-(4,5-dimethylthiazol-2-yl)-5-(3-carboxymethoxyphenyl)-2-(4-sulfophenyl)-2H-tetrazolium; MTT, 3-(4,5-dimethylthiazol-2-yl)-2,5-diphenyltetrazolium bromide; PI, propidium iodide; SB, sodium butyrate; Uro-A, urolithin A; X-gal, 5-bromo-4-chloro-3- indolyl-B-d-galactopyranoside.

### 3.3 Quality Assessment

The risk of bias assessment of the 15 included studies revealed an overall high methodological quality. Most studies (n = 13) were classified as Klimisch category 1, with scores ranging from 15 to 18, reflecting generally reliable results without restrictions. Two studies were classified as Klimisch Category 2, with scores between 11 and 14, indicating reliable findings with minor restrictions due to incomplete characterization and source information of the tested substances, as well as partial reporting of certain study parameters. Despite these minor limitations, these studies were considered in this review to integrate all available evidence. Common methodological gaps included the widespread absence of positive controls and an incomplete description of outcome measurements. Accordingly, the overall body of evidence can be considered moderate to high quality, supporting the reliability of the findings while acknowledging minor limitations in specific studies (Supplementary Table S1).

### 3.4 The Anticancer Potential of Uro-A in CRC

#### 3.4.1 Antiproliferative Activity

This section reviews the *in vitro* evidence of the antiproliferative effects of Uro-A in CRC cell lines and the non-tumorigenic CCD18-Co cell line, highlighting the experimental conditions and key outcomes. Table 3 provides a summary of these effects reported in the selected studies. For each reference, the table specifies the cell lines tested, compounds concentrations, exposure times, experimental methods employed, and main outcomes. Building on this, Table 4 offers an overview of the range of effective concentrations and half-maximal inhibitory concentrations (IC_50_) values for Uro-A monotherapy across all studies for each cell line, highlighting their corresponding relative sensitivities.

**Table 3:** Antiproliferative effects of Uro-A in human colorectal cell lines

Ref.	Human cell line	Concentration	Exposure time	Assay	Outcome
[20]	Caco-2	50 and 100 μM Uro-A	24–48 h	Trypan blue exclusion; MTT	Dose- and time-dependent ↓ in proliferation; most sensitive
	SW480	50 and 100 μM Uro-A	24–48 h	Trypan blue exclusion; MTT	Dose- and time-dependent ↓ in proliferation; intermediate sensitivity
	HT-29	50 and 100 μM Uro-A	24–48 h	Trypan blue exclusion; MTT	Dose-dependent and time-independent ↓ in proliferation; most resistant, particularly at 50 μM after 48 h
[22]	Caco-2	0.8–400 μM Uro-A	48–72 h	MTT	Time-dependent ↓ in proliferation; most sensitive; IC_50_ = 42.80 μM (48 h); IC_50_ = 32.50 μM (72 h)
	SW480	0.8–400 *μ*M Uro-A	48–72 h	MTT	Time-dependent ↓ in proliferation; intermediate sensitivity; IC_50_ = 46.01 μM (48 h); IC_50_ = 35.92 μM (72 h)
	HT-29	0.8–400 μM Uro-A	48–72 h	MTT	Time-dependent ↓ in proliferation; most resistant; IC_50_ = 59.45 μM (48 h); IC_50_ = 49.92 μM (72 h)
[23]	Caco-2	100 μM Uro-A	48 h	Trypan blue exclusion	Strong ↓ in proliferation; most sensitive
	SW480	100 μM Uro-A	48 h	Trypan blue exclusion	Strong ↓ in proliferation (slightly lower than Caco-2); intermediate sensitivity
	HT-29	100 μM Uro-A	48 h	Trypan blue exclusion	Moderate ↓ in proliferation; intermediate sensitivity
	CCD18-Co	100 μM Uro-A	48 h	Trypan blue exclusion	Low ↓ in cell proliferation; most resistant
[15]	HT-29	1.5, 3, 6, 12, 25, 50, 100 and 200 μM Uro-A	24–48 h	AlamarBlue	Dose- and time-dependent ↓ in proliferation; most sensitive; IC_50_ = 30.762 μM
	SW480	1.5, 3, 6, 12, 25, 50, 100 and 200 μM Uro-A	24–48 h	AlamarBlue	Dose- and time-dependent ↓ in proliferation; intermediate sensitivity; IC_50_ = 39.354 μM
	SW620	1.5, 3, 6, 12, 25, 50, 100 and 200 μM Uro-A	24–48 h	AlamarBlue	Dose- and time-dependent ↓ in proliferation; most resistant; IC_50_ = 59.517 μM
	HT-29	3.125–200 μM Uro-A	24–48 h	MTT	Dose-dependent ↓ in viability; most sensitive; IC_50_ = 25.456 μM
[29]	SW480	3.125–200 μM Uro-A	24–48 h	MTT	Dose-dependent ↓ in viability; intermediate sensitivity; IC_50_ = 38.135 μM
	SW620	3.125–200 μM Uro-A	24–48 h	MTT	Dose-dependent ↓ in viability; most resistant; IC_50_ = 53.561 μM
[21]	HT-29	5, 10, and 30 μg/mL	24–48 h	MTT	Time-independent ↓ in proliferation at 30 μg/mL manner
[25]	Caco-2	50 and 100 μM Uro-A	24–48 h	Trypan blue exclusion; MTT	Dose- and time-dependent ↓ in proliferation; most sensitive; IC_50_ = 95.9 μM (24 h); IC_50_ = 49.2 μM (48 h)
	CCD18-Co	50 and 100 μM Uro-A	24–48 h	Trypan blue exclusion; MTT	Significant ↓ in proliferation only at 100 μM or after 48 h; most resistant; IC_50_ = 148.0 μM (24 h); IC_50_ = 118.1 μM (48 h)
[26]	SW620	0, 0.15, 1.5 and 15 μM Uro-A	24 h	MTT	Dose-dependent ↓ in proliferation
[28]	*wt* HCT-116	0–60 μM Uro-A	48–72 h	Crystal violet staining	↓ In proliferation by more than 50%; IC_50_ = 39.2 μM (48 h); IC_50_ = 19.6 μM (72 h); most sensitive
	p53-/- HCT-116	0–60 μM Uro-A	48–72 h	Crystal violet staining	↓ In proliferation; IC_50_ = 38.4 μM (72 h); most resistant
[31]	HT-29	3.125, 6.25, 12.5, 25, 50, 100, and 200 μg/mL Uro-A	24 h	MTT	Dose-dependent ↓ in proliferation; IC_50_ = 120.4087 μg/mL
[12]	HT-29	20–100 μM Uro-A	24 h	MTT	↓ In proliferation by 20%; most resistant
	SW480	20–100 μM Uro-A	24 h	MTT	↓ In proliferation by 50%; most sensitive; IC_50_ = 50 μM
	SW620	20–100 μM Uro-A	24 h	MTT	↓ In proliferation by 50%; most sensitive; IC_50_ = 50 μM
	HCT-116	20–100 μM Uro-A	24 h	MTT	↓ In proliferation by 25%; intermediate sensitivity
[23]	Caco-2	MPhA (85 μM Uro-A, 10 μM Uro-C and 5 μM EA)	24–48 h	Trypan blue exclusion	Time-dependent ↓ in proliferation by 42.2% and 65.4% after 24 and 48 h, respectively
		MPhB (50 μM IsoUro-A, 30 μM Uro-A, 10 μM Uro-B, 5 μM Uro-C and 5 μM EA)	24–48 h	Trypan blue exclusion	Time-dependent ↓ in proliferation by 43.6% and 58.45% after 24 and 48 h, respectively
	CCD18-Co	MPhA (85 μM Uro-A, 10 μM Uro-C and 5 μM EA)	24–48 h	Trypan blue exclusion	↓ In proliferation by 16.4% and 31.9% after 24 and 48 h, respectively
		MPhB (50 μM IsoUro-A, 30 μM Uro-A, 10 μM Uro-B, 5 μM Uro-C and 5 μM EA)	24–48 h	Trypan blue exclusion	↓ In proliferation by 28.4% only at 48 h
[22]	Caco-2	Uro-A (10, 20, 50 and 100 μM) + 5-FU (0.2–400 μM)	48–72 h	MTT	Dose- and time-dependent ↓ in proliferation through an additive effect (CI = 0.98); intermediate sensitivity; IC_50_ = [15.08; 36.55] (48 h); IC_50_ = [13.00; 25.82] (72 h)
		Uro-A (10, 20, 50 and 100 μM) + 5^′^-DFUR (0.2–400 μM)	48–72 h	MTT	Dose- and time-dependent ↓ in proliferation through an additive effect (CI = 1.13); intermediate sensitivity; IC_50_ = [72.41; 80.14] (48 h); IC_50_ = [49.70; 62.83] (72 h)
	SW480	Uro-A (10, 20, 50 and 100 μM) + 5-FU (0.2–400 μM)	48–72 h	MTT	Dose- and time-dependent ↓ in proliferation through an additive effect (CI = 0.94); most sensitive; IC_50_ = [4.37; 6.25] (48 h); IC_50_ = [1.99; 3.08] (72 h)
		Uro-A (10, 20, 50 and 100 μM) + 5^′^-DFUR (0.2–400 μM)	48–72 h	MTT	Dose- and time-dependent ↓ in proliferation through an additive effect (CI = 1.00); most sensitive; IC_50_ = [57.04; 67.57] (48 h); IC_50_ = [19.77; 25.62] (72 h)
	HT-29	Uro-A (10, 20, 50 and 100 μM) + 5-FU (0.2–400 μM)	48–72 h	MTT	Dose- and time-dependent ↓ in proliferation through an additive effect (CI = 1.03); most resistant; IC_50_ = [60.18; 68.13] (48 h); IC_50_ = [26.83; 33.93] (72 h)
		Uro-A (10, 20, 50 and 100 μM) + 5^′^-DFUR (0.2–400 μM)	48–72 h	MTT	Dose- and time-dependent ↓ in proliferation through an additive effect (CI = 1.06); most sensitive; most resistant; IC_50_ = [162; 173.15] (48 h); IC_50_ = [92.17; 139.41] (72 h)
	HCT-116 (parental)	0, 1, 10, 25, and 50 μM Uro-A + 25 and 50 μM 5-FU	24–72 h	MTT; Flow cytometry	Dose- and time-dependent ↓ in proliferation through a synergistic effect; results corroborated by ↓ Ki-67^+^ cells
[30]	SW480 (parental)	0, 1, 10, 25, and 50 μM Uro-A + 25 and 50 μM 5-FU	24–72 h	MTT; Flow cytometry	Dose- and time-dependent ↓ in proliferation through a synergistic effect; results corroborated by ↓ Ki-67^+^ cells
	HCT-116-FUR	0, 1, 10, 25, and 50 μM Uro-A + 25 and 50 μM 5-FU	24–72 h	MTT; Flow cytometry	Dose- and time-dependent ↓ in proliferation through a synergistic effect; results corroborated by ↓ Ki-67^+^ cells
	SW480-FUR	0, 1, 10, 25, and 50 μM Uro-A + 25 and 50 μM 5-FU	24–72 h	MTT; Flow cytometry	Dose- and time-dependent ↓ in proliferation through a synergistic effect; results corroborated by ↓ Ki-67^+^ cells
[28]	*wt* HCT-116	1.25, 2.5, 5, 10 and 15 μM Uro-A + oxaliplatin	48–72 h	Crystal violet staining	↓ In proliferation through a synergistic effect (CI = [0.66–0.82]); most sensitive
	p53-/- HCT-116	2.5, 5, 10, 15, 20 and 30 μM Uro-A + oxaliplatin	72 h	Crystal violet staining	↓ In proliferation through a synergistic effect (CI = [0.92–0.94]); effect ↓ at >10 μM Uro-A (slight antagonism; CI = [1.09–1.21]); most resistant
[27]	HT-29	0.1, 1, 10, 25, 50 and 100 μM Uro-A	72 h	MTS	↓ In proliferation; IC_50_ = 43.9 μM
		50, 100, 500 μM and 1, 5, and 10 mm SB	72 h	MTS	↓ In proliferation; IC_50_ = 3 mM
		25 μM Uro-A + 2.5 mm SB	72 h	MTS	↓ In proliferation through an additive effect; results corroborated by ↓ PCNA
	HCT-116	0.1, 1, 10, 25, 50 and 100 μM Uro-A	72 h	MTS	↓ In proliferation; IC_50_ = 59.2 μM
		50, 100, 500 μM and 1, 5, and 10 mm SB	72 h	MTS	↓ In proliferation; IC_50_ = 0.7 mM
		50 μM Uro-A + 0.5 SB	72 h	MTS	↓ In proliferation through an additive effect, results corroborated by ↓ PCNA

Note: 5^′^-DFUR, 5^′^-Deoxy-5-fluorouridine; 5-FU, 5-Fluorouracil; CI, combination index; CRC, colorectal cancer; EA, ellagic acid; FUR, 5-Fluorouracil chemoresistant; h, hours; IC_50_, half-maximal inhibitory concentration; IsoUro-A, isourolithin A; Ki-67, marker of proliferation; MPhA, mixtures of ET metabolites mimicking metabotype A; MPhB, mixtures of ET metabolites mimicking metabotype B; MTS, 3-(4,5-dimethylthiazol-2-yl)-5-(3-carboxymethoxyphenyl)-2-(4-sulfophenyl)-2H-tetrazolium; MTT, 3-(4,5-dimethylthiazol-2-yl)-2,5-diphenyltetrazolium bromide; p53, tumor protein p53; PCNA, proliferating cell nuclear antigen; Ref., reference; SB, sodium butyrate; Uro-A, urolithin A; Uro-B, urolithin B; Uro-C, urolithin C; *wt*, wild type; ↓, decrease.

**Table 4:** Summary of the range of effective concentrations and IC_50_ values for Uro-A monotherapy reported across all studies for each cell line, with corresponding relative sensitivity

Human cell line	Effective concentrations/IC_50_	Relative sensibility	Observations
HCT-116	0–100 μM; IC_50_ ≈ 19.6–59.2 μM	High	Dose- and time-dependent
SW480	0.8–400 μM; IC_50_ ≈ 35.9–50.0 μM	Moderate	Dose- and time-dependent
Caco-2	0.8–400 μM; IC_50_ ≈ 32.5–95.9 μM	Moderate	Dose- and time-dependent
HT-29	0.8–400 μM; IC_50_ ≈ 25.46–59.45 μM	Moderate	Highly variable
SW620	1.5–200 μM; IC_50_ ≈ 50–59.5 μM	Low	Dose- and time-dependent, but overall weaker than the other cells
CCD18-Co	50–100 μM; IC_50_ ≈ 118–148 μM	Very low	Minimal effects at <100 μM

Note: IC_50_, half-maximal inhibitory concentration; Uro-A, urolithin A.

Uro-A Monotherapy

González-Sarrías et al. (2014) investigated the effects of Uro-A on the proliferation of Caco-2, HT-29, and SW480 colon cancer cell lines, through both trypan blue exclusion and 3-(4,5-dimethylthiazol-2-yl)-2,5-diphenyltetrazolium bromide (MTT) assays. Treatment with 50 and 100 μM of Uro-A for 24 and 48 h significantly decreased cell viability in all cell lines compared to control cells. The growth inhibition was more pronounced in Caco-2 cells, followed by SW480 cells, in a dose- and time-dependent manner. HT-29 cells proved to be the least sensitive to Uro-A, particularly at a concentration of 50 μM and after 48 h of treatment [20].

These findings are consistent with those of another study developed by González-Sarrías et al. (2015), in which they tested a broader concentration range of Uro-A (0.8–400 μM) in the same cell models over 48 and 72 h, via MTT assay. The results, expressed as IC_50_, confirmed a significant time-dependent antiproliferative effect in all cells with IC_50_ values below 50 μM. Once more, Caco-2 and HT-29 cells exhibited the greatest and least inhibition, respectively [22].

A similar study by González-Sarrías et al. (2016), using the trypan blue exclusion assay, revealed that treatment with 100 μM of Uro-A for 48 h led to a significant reduction in cell viability, with the effect being more pronounced in the Caco-2 and SW480 cell lines, followed by HT-29 and CCD18-Co cells, respectively [23].

Research designed by El-Wetidy et al. (2024) assessed the antiproliferative potential of Uro-A (1.5, 3, 6, 12, 25, 50, 100 and 200 μM) using an AlamarBlue test on HT-29, SW480, and SW620 cells for 24 and 48 h. The metabolite produced a concentration- and time-dependent decrease in cell growth, with IC_50_ values of 30.762 μM for HT-29, 39.354 μM for SW480 and 59.517 μM for SW620 [15].

In an earlier study, El-Wetidy et al. (2021) investigated different concentrations of Uro-A (3.125–200 μM) via MTT assay for 24 and 48 h. The viability of HT-29, SW480 and SW620 cells was significantly reduced in a dose-dependent manner, as corroborated by IC_50_ values of 25.456, 38.135 and 53.561 μM, respectively [29].

Cho et al. (2015) also determined the impact of Uro-A on the HT-29 cell line at 5, 10, and 30 μg/mL over 24–48 h, using MTT assays. At the highest concentration, Uro-A was able to inhibit cell proliferation regardless of the exposure time [21].

A study carried out by González-Sarrías et al. (2017) compared the response of Caco-2 cells and non-tumorigenic colon CCD18-Co cells after exposure to 50 and 100 μM of Uro-A for 24 to 48 h through both trypan blue exclusion and MTT assays. Both lines exhibited a concentration- and time-dependent suppression of cell growth, although the cancer cells were more sensitive to the treatment (IC_50_ = 95.9 μM (24 h); IC_50_ = 49.2 μM (48 h)) under all conditions tested. In CCD18-Co cells, this effect was only observed at the highest dose or after 48 h (IC_50_ = 148.0 μM (24 h); IC_50_ = 118.1 μM (48 h)) [25].

Zhao et al. (2018) monitored the behavior of the SW620 cell line following treatment with increasing doses of Uro-A (0, 0.15, 1.5 and 15 μM) via MTT assay. After 24 h, a significant inhibition of cell viability was observed, which was directly proportional to the concentration of Uro-A [26].

Similarly, Norden et al. (2019) assessed the response of both *wild type (wt)*—with functional tumor protein p53 (p53)—and p53-/- HCT-116 cell line to Uro-A via crystal violet staining assay. Within a concentration range of 0–60 μM, the metabolite decreased cell growth by over 50% in a dose- and time-dependent manner in *wt* cells, with IC_50_ values of 39.2 and 19.6 μM at 48 and 72 h, respectively. On the other hand, a less pronounced decrease was observed in p53-/- cells, with an IC_50_ of 38.4 μM at 72 h [28].

Tiwari et al. (2024) treated HT-29 cells with 3.125, 6.25, 12.5, 25, 50, 100, and 200 μg/mL of Uro-A for 24 h using MTT assay. The results confirmed a statistically significant suppression of cell proliferation in a dose-dependent manner, with IC_50_ values of 120.4087 μg/mL [31].

Lin et al. (2023) tested the effect of 20–100 μM of Uro-A on HT-29, SW480, SW620, and HCT-116 cell lines for 24 h. The MTT assay revealed a 50% reduction in cell viability in SW480 and SW620 cells (IC_50_ = 50 μM), 25% in HCT-116 cells, and approximately 20% in HT-29 cells. Thus, HT-29 cells proved to be the most resistant to the treatment, SW480 and SW620 presented the highest sensitivity and HCT-116 showed an intermediate response [12].

Overall, the antiproliferative effects of Uro-A varied considerably across the tested human cell lines. HCT-116 cells exhibited the highest sensitivity, with IC_50_ values ranging from 19.6 to 59.2 μM and a clear dose- and time-dependent response. SW480 and Caco-2 and cells showed moderate sensitivity, with IC_50_ values between 35.9–50.0 μM and 32.5–95.9 μM, respectively, also displaying dose- and time-dependent inhibition. HT-29 cells were moderately to poorly sensitive, with IC_50_ values of 25.46–59.45 μM and highly variable responses, indicating heterogeneous susceptibility. SW620 cells demonstrated low sensitivity (IC_50_ ≈ 50–59.5 μM), with dose- and time-dependent effects that were overall weaker compared to the other CRC lines. In contrast, non-tumorigenic CCD18-Co cells were markedly less responsive, with IC_50_ values of 118–148 μM and minimal growth inhibition observed at concentrations below 100 μM. These results suggest that Uro-A selectively inhibits the proliferation of CRC cells, with efficacy strongly dependent on cell line–specific characteristics (Table 4).

Combined Therapy with Uro-A, MPhA and MPhB

To explore how interindividual variability of CRC patients affects the biological activity of Uros, a subsequent study was performed by González-Sarrías et al. (2016) using MPhA (85 μM Uro-A, 10 μM Uro-C and 5 μM EA) and MPhB (50 μM IsoUro-A, 30 μM Uro-A, 10 μM Uro-B, 5 μM Uro-C and 5 μM EA), on the Caco-2 and CCD18-Co cells, over 24 to 48 h. Based on the trypan blue exclusion assay, both mixtures led to a significant inhibition of cellular proliferation in a dose- and time-dependent manner in Caco-2 cells. Specifically, MPhA induced a decrease of 42.2% at 24 h and 65.4% at 48 h, whereas MPhB caused 43.6% and 58.5% reductions, respectively. In CCD18-Co cells, both mixtures exhibited statistically significant effects at 48 h (MPhA: 31.9% inhibition; MPhB: 28.4% inhibition), although less pronounced. Unlike MPhB, this effect was also significant at 24 h with MPhA treatment (16.4%) [23].

Combined Therapy of Uro-A and Chemotherapeutic Agents

González-Sarrías et al. (2015) further explored the response of Caco-2, SW480 and HT-29 cell lines to the combined treatment of Uro-A and the chemotherapeutic agents 5-FU and 5^′^-DFUR via MTT assay. The authors tested different physiologically relevant concentrations of the metabolite (10, 20, 50 and 100 μM) together with graded doses of 5-FU and 5^′^-DFUR (0.2–400 μM) for 48 and 72 h. The findings indicated that co-treatment with Uro-A significantly improved the effectiveness of both drugs compared to their use alone through an additive effect, and especially when associated with 5-FU, in a dose- and time-dependent manner. Moreover, this outcome led to a greater decrease in the proliferation of SW480 cells followed by Caco-2 and HT-29 cells [22].

Ghosh et al. (2022) expanded the potential of the co-treatment by applying it to the parental HCT-116 and SW480 cells, and 5-FU chemoresistant (FUR) cell models, HCT-116-FUR and SW480-FUR. Using Uro-A at concentrations of 0, 1, 10, 25, and 50 μM in combination with 25 and 50 μM of 5-FU, over 24 to 72 h, they observed a statistically significant dose- and time-dependent inhibition of cell proliferation in both parental and resistant cell lines, with no significant differences observed within each group, as shown by MTT assay. This suggests that this compound acts as a chemosensitizer to 5-FU treatment, exerting a synergistic effect, since neither treatment alone induced a significant inhibition of cell proliferation. These findings were confirmed by flow cytometry with the reduction of marker of proliferation (Ki-67) positive cells in all cell lines [30].

Building upon on this approach, Norden et al. (2019) studied the combined use of Uro-A (1.25, 2.5, 5, 10 and 15 μM) and oxaliplatin, a standard chemotherapeutic agent used in CRC treatment, on *wt* and p53-/- HCT-116 cells over 48 to 72 h via crystal violet staining assay. Their results showed that Uro-A (1.25, 2.5, 5, 10 and 15 μM of Uro-A) potentiated the action of oxaliplatin compared to its use alone, indicating a synergistic interaction between the two compounds in *wt* cells. However, in p53-/- cells exposed to the co-treatment (2.5, 5, 10, 15, 20 and 30 μM Uro-A + oxaliplatin), this synergistic effect was reduced, and even a slight antagonism was observed at higher Uro-A concentrations (>10 μM) [28].

The co-treatments of Uro-A with conventional chemotherapeutic agents highlighted variable magnitudes of antiproliferative effects depending on both the cell line and the drug combination. When combined with 5-FU, Uro-A induced qualitative synergistic effects in HCT-116 cells, whereas additive interactions were observed in Caco-2, SW480, and HT-29 cells, as reflected by the combination index (CI), where CI < 1 indicates synergism, CI = 1 additivity, and CI > 1 antagonism (CI = 0.98 for Caco-2, 0.94 for SW480, and 1.03 for HT-29). Among these three cell lines, sensitivity followed the gradient SW480 > Caco-2 > HT-29, with HT-29 being the most resistant, as suggested by the CI slightly above 1 (Table 3).

Similarly, the Uro-A + 5^′^-DFUR combination yielded similar responses, with CI values ranging from 1.00 to 1.13 across SW480, HT-29, and Caco-2 cells. Again, SW480 cells exhibited the highest sensitivity, while HT-29 cells were the most resistant. When comparing both treatments, Uro-A + 5-FU showed stronger cytotoxic effects and lower IC_50_ values, suggesting a more potent interaction than Uro-A + 5^′^-DFUR (Table 3).

In contrast, the combination of Uro-A with oxaliplatin evidenced differential sensitivity patterns among HCT-116 sublines. *wt* cells exhibited synergistic interactions (CI = 0.66–0.82), whereas p53-/- cells displayed reduced synergy or slight antagonism (CI = 0.92–1.21), confirming that p53 deficiency confers greater chemoresistance (Table 3).

Combined Therapy of Uro-A and Sodium Butyrate

Tortora et al. (2018) performed a study in which they tested Uro-A and SB in HT-29 and HCT-116 colon cancer cell lines, building on the positive outcomes of their previous *in vivo* study on preneoplastic microscopic lesions. Throughout 72 h, these metabolites were administered individually (0.1, 1, 10, 25, 50 and 100 μM of Uro-A; 50, 100, 500 μM, 1, 5, and 10 mM of SB) or in combined treatments (25 or 50 μM of Uro-A + 500 μM, 1 mm and 5 mm of SB) in both cells. The results of 3-(4,5-dimethylthiazol-2-yl)-5-(3-carboxymethoxyphenyl)-2-(4-sulfophenyl)-2H-tetrazolium (MTS) viability assay confirmed that Uro-A and SB, when administered separately, exerted an antiproliferative effect on HT-29 cells, with IC_50_ values of 43.9 μM and 3 mm, and on HCT-116 cells, with IC_50_ values of 59.2 μM and 0.7 mm, respectively. A statistically significant decrease in proliferating cell nuclear antigen (PCNA) was observed in the cells treated with SB alone, whereas only a slight decrease was detected under co-treatment with Uro-A. At a fixed concentration of Uro-A (25 μM for HT-29; 50 μM for HCT-116) and variable concentrations of SB (500 μM, 1 and 5 mm), the IC_50_ value of SB did not change significantly. However, at sub-IC_50_ concentrations of SB (2.5 mm for HT-29; 0.5 mm for HCT-116), the inhibition of cellular proliferation was potentiated through an additive effect [27].

#### 3.4.2 Impact on Colonosphere Formation and Cancer Stem Cells Markers

Colonospheres are three-dimensional structures used to study the behavior of colorectal cancer stem cells (CSC), which have the ability to form floating spheroids *in vitro* in a serum-free medium supplemented with growth factors [32].

An *in vitro* study conducted by Nuñez-Sánchez et al. (2016) examined the effects of MPhA and MPhB on the ability of Caco-2 cells to form colonospheres enriched with CSC. A low (C_1_) and high (C_2_) concentration of each mixture was tested:  = 0.85 μM Uro-A + 0.1 μM Uro-C + 0.05 μM EA;  = 17 μM Uro-A + 2 μM Uro-C + 1 μM EA;  = 0.3 μM Uro-A + 0.5 μM IsoUro-A + 0.1 μM Uro-B + 0.05 μM Uro-C + 0.05 μM EA;  = 6 μM Uro-A + 10 μM IsoUro-A + 2 μM Uro-B + 1 μM Uro-C + 1 μM EA. The results demonstrated a significant decrease in the number and size of the colonospheres at the highest concentration, with this effect being more pronounced with the MPhB treatment [24].

Furthermore, the influence of both mixtures on the expression of the stem cells (SC) markers aldehyde dehydrogenase (ALDH) and prominin-1 (CD133) was evaluated by Aldefluor assay and flow cytometry, respectively. The data obtained revealed that treatment with C_2_ MPhA significantly reduced the subpopulation of cells with high ALDH (ALDH^high^) activity, whereas MPhB did not cause any changes. However, none of the treatments had any impact on CD133 expression [24].

#### 3.4.3 Anticlonogenic Growth

A research by Giménez-Bastida et al. (2020) analyzed the clonogenic capacity of the Caco-2, HCT-116, and HT-29 cells, as well as the non-tumoral CCD18-Co cell line, using a clonogenic assay in which 0.5, 1, and 10 μM of Uro-A, MPhA (80% Uro-A + 20% Uro-C) and MPhB (50% IsoUro-A + 20% Uro-B + 20% Uro-A + 10% Uro-C) were administered. After 14 days, only the highest concentration of each treatment significantly inhibited the formation of HCT-116 cell colonies. However, in Caco-2 cells this effect was observed at both 1 μM and 10 μM of Uro-A, MPhA and MPhB, with Uro-A being the most effective in both cell lines. In HT-29 cells only 10 μM of Uro-A exerted a significant anticlonogenic effect, while CCD18-Co cells demonstrated no changes in response to any of the treatments [13].

El-Wetidy et al. (2024) investigated the effect of increasing doses of Uro-A (25, 50, and 100 μM) on the colony formation capacity of the HT-29, SW480, and SW620 cell lines for 7 days through a clonogenic assay. Although 25 μM did not have a significant effect, 50 μM produced a statistically significant reduction in the number of colonies by 63.1%, 86.6%, and 87.9% in SW620, SW480, and HT-29 cells, respectively. The highest concentration of Uro-A caused almost total inhibition of clonogenic growth in all cell lines. Additionally, severe morphological changes were detected after treatment, including cell membrane contraction and the absence of crystal violet staining (HT-29), altered morphology (SW480), and cell membrane rupture (SW480 and SW620) [15].

#### 3.4.4 Induction of Cell Cycle Arrest

According to a study developed by González-Sarrías et al. (2014), treatment with Uro-A (50 and 100 μM) for 24 to 48 h significantly arrested the cell cycle at G_2_/M and S phases, in a dose- and time-dependent manner, in Caco-2 and SW480 cells, respectively, as determined by flow cytometry. On the other hand, HT-29 cells were more resistant to the treatment, showing a dose-dependent but time-independent response [20].

El-Wetidy et al. (2021) assessed the effects of different concentrations of Uro-A (25, 50, and 100 μM) on HT-29, SW480, SW620, and CCD18-Co cell lines over 24 to 48 h via propidium iodide (PI) staining and flow cytometry. The results showed a dose- and time-dependent cell cycle blockage at the G_2_/M phase, accompanied by a decrease in the G_0_/G_1_ population in HT-29, SW480, and SW620 cells. The most pronounced effect was observed in SW480 cells, where up to 96% of the cells accumulated at G_2_/M after 48 h. On the other hand, no significant changes were observed in CCD18-Co cells. To understand the mechanisms underlying cell cycle interruption, the authors evaluated the expression of the regulatory proteins cyclin B1 (G_2_/M phase) and cyclin-dependent kinase 6 (CDK6; G_1_/S phase) through western blot analysis in cancer cells. The data confirmed a significant dose-dependent overexpression of cyclin B1 and CDK6 in all cell lines [29].

It has also been found that Uro-A, at a concentration of 30 μg/mL, triggered a blockage of the HT-29 cells at the G_2_/M phase after 48 h, as demonstrated by Cho et al. (2015). To investigate whether this effect might be related to cyclin-dependent kinase inhibitor 1A (p21) overexpression, the authors performed its quantification via western blot. The results proved that Uro-A, under the tested conditions, produced a statistically significant upregulation of p21 [21].

In a study conducted by González-Sarrías et al. (2017), Caco-2 and CCD18-Co cells were treated with 50 and 100 μM of Uro-A for 24 to 48 h and analyzed by flow cytometry. In a dose- and time-dependent manner, cancer cells were significantly arrested in the S and G_2_/M phases, concomitant with a decrease in the distribution of cells in the G_0_/G_1_ phase. Although less pronounced, normal cells also exhibited G_2_/M phase arrest, with this effect being more significant with increasing concentration and exposure length [25].

Zhao et al. (2018) performed an experiment in which 0, 1.5, 15 or 30 μM of Uro-A were administered to the SW620 cell line for 24 h using flow cytometry. The reported findings demonstrated significant cell cycle blockage at G_2_/M phase, compatible with an increase in cancer cells at this stage [26].

Norden et al. (2019) investigated the effects of 20 and 40 μM of Uro-A on *wt* and p53-/- HCT-116 cells over 24 and 48 h using flow cytometry. The results revealed a significant cell cycle arrest at the G_2_/M phase in both cell types. After 48 h of treatment with the higher concentration, 47% and 55% of p53-/- and *wt* cells, respectively, were in the G_2_/M phase [28].

In the experiment performed by González-Sarrías et al. (2016), exposure of Caco-2 and SW480 cells to Uro-A (100 μM; 48 h) resulted in a pronounced accumulation of cells in the S and G_2_/M phases of the cell cycle, whereas HT-29 and CCD18-Co cells displayed cell cycle blockage only at the G_2_/M, under the same experimental conditions, as determined by flow cytometry. In the same study, Caco-2 cells treated with MPhA (85 μM Uro-A, 10 μM Uro-C, and 5 μM EA) and MPhB (50 μM IsoUro-A, 30 μM Uro-A, 10 μM Uro-B, 5 μM Uro-C, and 5 μM EA) also exhibited significant cell cycle arrest at the S and G_2_/M phases after 24 and 48 h. In contrast, in CCD18-Co cells this effect was only significant after 48 h, with a slight blockage in the G_2_/M phase [23].

Giménez-Bastida et al. (2020) assessed the impact of Uro-A, MPhA (80% Uro-A + 20% Uro-C) and MPhB (50% IsoUro-A + 20% Uro-B + 20% Uro-A + 10% Uro-C) for 5 days on HCT-116, Caco-2, HT-29 and CCD18-Co cell lines through flow cytometry. Treatment with 10 μM of Uro-A and MPhA led to a significant HCT-116 cells accumulation at the G_2_/M phase, as well as a reduction in the G_0_/G_1_ and S phases. Furthermore, the same dose of Uro-A, MPhA and MPhB caused a statistically significant G_2_/M phase arrest in Caco-2 cells. In contrast, HT-29 and CCD18-Co cells were not sensitized by the treatment, and no significant changes were observed [13].

González-Sarrías et al. (2015) investigated the effects of Uro-A (10 and 20 μM) treatment alone or in combination with 5-FU or 5^′^-DFUR on the cell cycle distribution of Caco-2, SW480 and HT-29 cells for 48 h by flow cytometry. Different concentrations of the chemotherapeutic agents were used depending on the cell line: 10 μM 5-FU and 50 μM 5^′^-DFUR for SW480 cells, and 50 μM 5-FU and 100 μM 5^′^-DFUR for Caco-2 and HT-29 cells. The results showed that Caco-2 and SW480 cells accumulated significantly and progressively at the G_2_/M phase at the highest concentration of Uro-A, while 5-FU and 5^′^-DFUR significantly increased the proportion of cells in the S phase. Furthermore, both combined treatments with Uro-A significantly arrested cell cycle at S phase in all cell lines, and at the G_2_/M phase specifically in Caco-2 and SW480 cells. This dual-phase blockade suggests an additive rather than a synergistic effect between Uro-A and the chemotherapeutic agents [22].

Additionally, the authors explored the underlying mechanisms of cell cycle interruption, performing western blot analysis after 48 h of treatment with each compound (alone or combined), and found that 20 μM of Uro-A led to a significant increase in the expression of cyclins A (S/G_2_ phase) and B1 (G_2_/M phase) in Caco-2 and SW480 cells. On the other hand, 5-FU and 5^′^-DFUR treatments induced a significant increase in both cyclins in all cells. Finally, co-treatment with Uro-A and the two chemotherapeutic agents caused a small increase in both cyclins, compared to single treatments, which was particularly significant in Caco-2 cells with cyclin B1, and in SW480 cells with cyclin A and B1, when Uro-A + 5^′^-DFUR were administered [22].

#### 3.4.5 Induction of Apoptosis

This section reviews the *in vitro* evidence of the apoptosis-inducing potential of Uro-A in CRC cell lines and the non-tumorigenic CCD18-Co cell line, highlighting the experimental conditions and key outcomes. Table 5 provides a summary of these effects reported in the selected studies. For each reference, the table specifies the cell lines tested, compounds concentrations, exposure times, experimental methods employed, and main outcomes.

**Table 5:** Apoptosis-inducing potential of Uro-A in human colorectal cell lines

Ref.	Human cell line	Concentration	Exposure time	Assay	Outcome
[21]	HT-29	30 μg/mL Uro-A	48 h	Annexin V-FITC/PI	↑ In early and late apoptotic cells (early > late)
				JC-1	Mitochondrial membrane potential depolarization
				Colorimetric caspase protease assay	↑ In caspase-8 and caspase-9; ↑ caspase-3 activity (small effect size)
				Western blot	↑ PARP cleavage (small effect size)
[22]	Caco-2	20 μM Uro-A; 50 μM 5-FU; 100 μM 5^′^-DFUR; Uro-A + 5-FU; Uro-A + 5^′^-DFUR	48 h	Annexin V/PI; Hoechst 33242	5-FU: ↑ early and late apoptosis; 5^′^-DFUR and Uro-A: not significant; Uro-A + 5-FU: slight ↑, not significant; Uro-A + 5^′^-DFUR: ↑ in early apoptosis
				Flow cytometry	5-FU: ↑ caspase-8 and 9 (especially caspase-9); Uro-A: small ↑ (not significant) of both caspases; co-treatments: slight ↑ of both caspases (not significant); Uro-A + 5^′^-DFUR: ↑ caspase-9
	HT-29	20 μM Uro-A; 50 μM 5-FU; 100 μM 5^′^-DFUR; Uro-A + 5-FU; Uro-A + 5^′^-DFUR	48 h	Annexin V/PI; Hoechst 33242	5-FU and 5^′^-DFUR: ↑ in early apoptosis; Uro-A: not significant; co-treatments: not significant
				Flow cytometry	5-FU: ↑ caspase-8 and 9; 5^′^-DFUR: ↑ caspase-8 and 9; Uro-A: not significant; co-treatments: slight ↑ in both caspases (not significant)
	SW480	20 μM Uro-A; 10 μM 5-FU; 50 μM 5^′^-DFUR; Uro-A + 5-FU; Uro-A + 5^′^-DFUR	48 h	Annexin V/PI; Hoechst 33242	5-FU: ↑ early and late apoptosis; 5^′^-DFUR and Uro-A: not significant; co-treatments: slight ↑, not significant
				Flow cytometry	5-FU: ↑ caspase-8 and -9; 5^′^-DFUR: ↑ caspase-8; Uro-A: small ↑ in both caspases (not significant); co-treatments: slight ↑ in both caspases (not significant)
[23]	Caco-2	MPhA (85 μM Uro-A + 10 μM Uro-C + 5 μM EA); MPhB (50 μM IsoUro-A + 30 μM Uro-A + 10 μM Uro-B + 5 μM Uro-C + 5 μM EA)	24–48 h	Annexin V/PI	Small ↑ but time-dependent in early and late apoptotic cells after both treatments
	CCD18-Co	MPhA (85 μM Uro-A + 10 μM Uro-C + 5 μM EA); MPhB (50 μM IsoUro-A + 30 μM Uro-A + 10 μM Uro-B + 5 μM Uro-C + 5 μM EA)	24–48 h	Annexin V/PI	No significant apoptotic effect observed
[25]	Caco-2	50 and 100 μM Uro-A	24–48 h	Annexin V/PI	Dose- and time-dependent ↑ in early and late apoptotic cells
	CCD18-Co	50 and 100 μM Uro-A	24–48 h	Annexin V/PI	No significant apoptotic effect observed
[26]	SW620	0, 1.5, 15, and 30 μM Uro-A	24 h	Annexin V-FITC/PI	Only 30 μM Uro-A ↑ apoptosis; lower concentrations had no effect
[27]	HT-29	25 μM Uro-A; 2.5 mm SB, Uro-A + SB	24–72 h	Western blot	↑ Caspase-3 activity with co-treatment at 24 h, ↑ at 72 h but not significant
	HCT-116	50 μM Uro-A; 0.5 mm SB; Uro-A + SB	24–72 h	Western blot	
[29]	HT-29	25, 50 and 100 μM Uro-A	24–48 h	Annexin V/Pi	Dose- and time-dependent ↑ in late apoptosis; ↑ in early apoptosis only at 100 μM after 48 h
			48 h	Western blot	Dose-dependent ↓ in XIAP and Bcl-2; ↑ in p53, p21, cytochrome c, cleaved caspase-3 and -9
	SW480	25, 50 and 100 μM Uro-A	24–48 h	Annexin V/Pi	Similar pattern to HT-29, but more ↑ in early apoptosis
			48 h	Western blot	Dose-dependent ↓ in XIAP and Bcl-2; ↑ in p53, p21, cytochrome c, cleaved caspase-3 and -9
	SW620	25, 50 and 100 μM Uro-A	24–48 h	Annexin V/Pi	↑ in late apoptosis at 100 μM; lesser effects at lower doses
			48 h	Western blot	Dose-dependent ↓ in XIAP and Bcl-2; ↑ in p53, p21, cytochrome c, cleaved caspase-3 and -9
	CCD18-Co	25, 50 and 100 μM Uro-A	24–48 h	Annexin V/Pi	No significant apoptotic effect observed
[30]	HCT-116 (parental)	50 μM Uro-A; 50 μM of 5-FU; Uro-A + 5-FU	24 h	Annexin V-FITC/PI; flow cytometry	Uro-A: slight ↑ (not significant); 5-FU and Uro-A + 5-FU: ↑ apoptosis
	SW480 (parental)	50 μM Uro-A; 50 μM of 5-FU; Uro-A + 5-FU	24 h	Annexin V-FITC/PI; Flow cytometry	Uro-A: slight ↑ (not significant); 5-FU and Uro-A + 5-FU: ↑ apoptosis
	HCT-116-FUR	50 μM Uro-A; 50 μM of 5-FU; Uro-A + 5-FU	24 h	Annexin V-FITC/PI; flow cytometry	Only co-treatment ↑ apoptosis
			24 h	Western blot	Co-treatment ↑ cleaved caspase-9 and Bax expression
	SW480-FUR	50 μM Uro-A; 50 μM of 5-FU; Uro-A + 5-FU	24 h	Annexin V-FITC/PI; flow cytometry	Only co-treatment ↑ apoptosis

Note: 5^′^-DFUR, 5^′^-Deoxy-5-fluorouridine; 5-FU, 5-Fluorouracil; Annexin V/PI, annexin V/propidium iodide; Annexin V-FITC/PI, annexin V-fluorescein isothiocyanate/propidium iodide; Bax, B-cell leukemia/lymphoma 2 associated X protein; Bcl-2, B-cell leukemia/lymphoma 2 protein; CRC, colorectal cancer; EA, ellagic acid; FUR, 5-Fluorouracil chemoresistant; h, hours; IsoUro-A, isourolithin A; JC-1, 5,5^′^,6,6^′^-tetrachloro-1,1^′^,3,3^′^-tetraethyl-benzimidazolocarbocyanine iodide; MPhA, mixtures of ET metabolites mimicking metabotype A; MPhB, mixtures of ET metabolites mimicking metabotype B; p21, cyclin-dependent kinase inhibitor 1A; p53, tumor protein p53; PARP, poly (adenosine diphosphate-ribose) polymerase; Ref, reference; SB, sodium butyrate; Uro-A, urolithin A; Uro-B, urolithin B; Uro-C, urolithin C; XIAP, X-linked inhibitor of apoptosis protein; ↑, increase; ↓, decrease.

Cho et al. (2015) investigated the apoptotic mechanisms of 30 μg/mL of Uro-A in HT-29 cells over 48 h through an annexin V-fluorescein isothiocyanate/propidium iodide (annexin V-FITC/PI) assay. The metabolite induced a significant increase in both early and late apoptotic cells, with a more pronounced effect on early apoptosis. In order to confirm these findings, the authors evaluated the impact of Uro-A (5, 10, and 30 μg/mL) on mitochondrial membrane potential depolarization in HT-29 cells over a 24-h period using 5,5^′^,6,6^′^-tetrachloro-1,1^′^,3,3^′^-tetraethyl-benzimidazolocarbocyanine iodide (JC-1) dye assay. The treatment significantly altered mitochondrial membrane potential in a dose-dependent manner, which supports the involvement of the intrinsic pathway in Uro-A-induced apoptosis [21].

Subsequently, they examined the activity of initiator caspases 8 and 9 after treatment with 30 μg/mL of Uro-A for 48 h using a colorimetric caspase protease assay. The activity of both caspases increased significantly in the treated cells, which supports the involvement of both pathways in the induction of apoptosis mediated by Uro-A. The authors also investigated the activity of effector caspase 3, as well as the cleavage of poly (adenosine diphosphate ribose) polymerase (PARP) using western blot analysis. The findings demonstrated a statistically significant increase in caspase 3 activity and PARP cleavage after treatment, although the effect size was small [21].

In a study performed by González-Sarrías et al. (2015), the induction of apoptosis in Caco-2, SW480, and HT-29 cells was assessed after 48 h of treatment with Uro-A (20 μM), 5-FU or 5^′^-DFUR (10, 50 or 100 μM, depending on the specific IC_50_ value of each cell line), using the annexin V/propidium iodide (annexin V/PI) assay and Hoechst 33242 dye. In Caco-2 and SW480 cells, 5-FU produced a significant increase in both early and late apoptosis, whereas in HT-29 cells it only caused a rise in the number of early apoptotic cells. Notably, Uro-A alone did not induce a statistically significant apoptotic effect in any of the cell lines. Similar results occurred with 5^′^-DFUR, which only significantly increased the number of early apoptotic HT-29 cells. Although the co-treatments slightly enhanced Caco-2 and SW480 apoptotic cells, compared with either chemotherapeutic agents alone, these changes were not significant. However, a significant increase in early apoptotic Caco-2 cells occurred when treated with Uro-A + 5^′^-DFUR [22].

Additionally, the role of the intrinsic and extrinsic pathways of apoptosis was evaluated through flow cytometry for 48 h. Exposure to 5-FU led to a significant activation of caspase 8 and 9 activity in all cells, with this effect being considerably more pronounced in Caco-2 cells and in caspase 9. In contrast, 5^′^-DFUR only caused a significant increase in caspase activity in HT-29 cells (caspase 8 and 9) and in SW480 cells (caspase 8). Although not statistically significant, Uro-A also promoted a slight activation of both caspases in all cells except HT-29 cells. Compared with the individual treatments, the co-treatments produced a small increase in the activity of both caspases in all cells, though it was not considered significant. On the other hand, a statistically significant increase in caspase 9 was detected in Caco-2 cells treated with Uro-A + 5^′^-DFUR [22].

González-Sarrías et al. (2016) determined the percentage of early and late apoptotic Caco-2 and CCD18-Co cells after treatments with MPhA (85 μM Uro-A + 10 μM Uro-C + 5 μM EA) and MPhB (50 μM IsoUro-A + 30 μM Uro-A + 10 μM Uro-B + 5 μM Uro-C + 5 μM EA), using the annexin V/PI assay, over 24 and 48 h. The results revealed a slight increase in cancer apoptotic cells after each treatment in a time-dependent manner. On the other hand, the mixtures did not cause significant changes on normal cells [23].

After treating Caco-2 and CCD18-Co cells with 50 and 100 μM of Uro-A for 24 and 48 h, González-Sarrías et al. (2017) observed a statistically significant, time- and dose-dependent increase in the percentage of early and late apoptotic cells in cancer cells, as determined by an annexin V/PI assay. In contrast, no effects were observed on the CCD18-Co cell line [25].

Zhao et al. (2018) performed an annexin V-FITC/PI assay to evaluate the effect of 0, 1.5, 15, and 30 μM of Uro-A on the induction of apoptosis in SW620 cells over 24 h. The highest concentration induced apoptosis in cancer cells, unlike the other concentrations [26].

Another study by Tortora et al. (2018) examined the expression of caspase-3 using western blot in HT-29 cell line treated with 25 μM of Uro-A, 2.5 mm of SB, or both combined, as well as in HCT-116 cells treated with 50 μM of Uro-A, 0.5 mm of SB, or both combined. After 24 h, the co-treatment significantly increased the expression of the effector caspase in both cell lines. After 72 h, protein expression also increased, though not significantly [27].

El-Wetidy et al. (2021) developed a study in which they tested 25, 50 and 100 μM of Uro-A in HT-29, SW480, SW620 and CCD18-Co cells, for 24 and 48 h using annexin V/PI assay. In HT-29 cells, the metabolite produced a dose- and time-dependent increase in late apoptosis. Early apoptosis was significantly affected only after 48 h of treatment with 100 μM of the compound. Similar results were observed in SW480 cells, although with markedly enhanced early apoptosis. In SW620 cell line, the highest concentration of Uro-A exerted the greatest effect, especially in late apoptosis, while CCD18-Co cells remained unaffected [29].

The same authors further investigated changes in the expression of the proteins cytochrome c, p53, p21, X-linked inhibitor of apoptosis protein (XIAP), B-cell leukemia/lymphoma 2 protein (Bcl-2), caspase-3, and caspase-9 in cells treated with 25, 50, and 100 μM of Uro-A for 48 h. Western blot analysis revealed dose-dependent depletion of the anti-apoptotic proteins XIAP and Bcl-2, upregulation of p53 and p21, and overexpression of cytochrome c, cleaved caspase-3, and cleaved caspase-9 across all cancer cell lines [29].

Ghosh et al. (2022) assessed the effects of 50 μM of Uro-A and/or 50 μM of 5-FU in parental HCT-116 and SW480 cell lines, as well as in HCT-116-FUR and SW480-FUR cells. Apoptosis was assessed using annexin V-FITC/PI staining followed by flow cytometry after 24 h. Uro-A caused a slight increase in apoptosis in all cell lines, but it was not statistically significant. Both 5-FU and Uro-A + 5-FU significantly induced apoptosis in the parental cell lines. However, only the co-treatment effectively promoted apoptosis in both resistant cells. Additionally, through western blot analysis, the authors observed an overexpression of caspase-9, cleaved caspase-9, and B-cell leukemia/lymphoma 2 associated X protein (Bax) in HCT-116-FUR cells treated with the combination of Uro-A and 5-FU, compared to the individual treatments [30].

#### 3.4.6 Anti-Migratory and Anti-Invasive Effects

Zhao et al. (2018) evaluated the mobility and expression of matrix metalloproteinase-9 (MMP-9) in SW620 cells treated with 0.05 μM of Uro-A for 48 h, using chamber migration and colorimetric assays, respectively. The results revealed a significant reduction in the number of migratory cells, delay in cell migration, and decrease in MMP-9 activity by 35.2%, 21.3%, and 29.8%, respectively [26].

Ghosh et al. (2022) examined the effects of both monotherapy and co-treatment with 50 μM Uro-A and 50 μM 5-FU on cell migration and invasion, using standard scratch assays and three-dimensional invasion assays, respectively, in HCT-116-FUR and SW480-FUR cell lines. While the monotherapies did not cause any significant changes in the FUR cells, the co-treatment led to a significant decrease in cell migration and invasion in HCT-116-FUR and SW480-FUR cell lines [30].

To clarify how Uro-A + 5-FU exerts its anti-migratory effects, the authors determined the expression of epithelial-mesenchymal transition (EMT) mediators, including β-catenin and zinc finger protein SNAI1 (Snail) (EMT inducers), as well as zona occludens 1 (ZO1) and E-cadherin (EMT inhibitors). The results were assessed via western blot analysis, confocal imaging and messenger ribonucleic acid quantification in HCT-116-FUR and SW480-FUR cells after 24 h. In the absence of any treatment, both cell lines showed increased levels of β-catenin and Snail protein, as well as decreased levels of ZO1 and E-cadherin, compared to the parental HCT-116 and SW480 lines. On the other hand, co-treatment with 50 μM of Uro-A and 50 μM of 5-FU resulted in a significant overexpression of ZO1 and E-cadherin, along with a significant downregulation of β-catenin and Snail protein in both FUR cells [30].

A study conducted by El-Wetidy et al. (2024) tested 25 and 50 μM of Uro-A on HT-29, SW480, and SW620 cells aiming to evaluate its effects on cellular migration rate through a wound healing assay. After 24 and 48 h of treatment, the cellular migration rate decreased significantly in all cell lines compared to the control cells in a dose-dependent manner, with the effects being considerably superior in the HT-29 cell line, followed by SW480 and SW620 [15].

To understand the underlying mechanisms of this metastasis inhibition, the expression levels of MMP-1, MMP-2, and tissue inhibitor of metalloproteinases 1 (TIMP-1) were evaluated using western blot analysis for 48 h and after treatment with 0, 25, 50 and 100 μM of Uro-A. The results evidenced a statistically significant decrease in MMP-1 and MMP-2 levels, as well as an increase in TIMP-1 expression in HT-29, SW480, and SW620 cells [15].

#### 3.4.7 Induction of Cellular Senescence

Giménez-Bastida et al. (2020) investigated whether cellular senescence induction was linked to the previously observed anti-clonogenic effect and cell cycle arrest in Caco-2, HT-29, HCT-116, and CCD18-Co cells treated with 10 μM of Uro-A, MPhA (80% Uro-A + 20% Uro-C), and MPhB (50% IsoUro-A + 20% Uro-B + 20% Uro-A + 10% Uro-C). To this purpose, they evaluated the activity of senescence-associated β-galactosidase (SA-β-gal) over the period of 1, 3, and 5 days of treatment using the fluorogenic substrate 5-dodecanoylaminofluorescein di-β-D-galactopyranoside (FDG). Although the induction of cellular senescence was not observed in Caco-2, HT-29, and CCD18-Co cells, the enzyme exhibited significant and considerable activity in HCT-116 cells in a time-dependent manner after treatment with Uro-A and MPhA. To validate the results, a histochemical staining with 5-bromo-4-chloro-3-indolyl-β-D-galactopyranoside (X-gal) was performed at day 5, revealing an increase in blue-stained cells indicative of SA-β-gal activity in HCT-116 cell line treated with Uro-A and MPhA. The remaining cell lines were used as negative controls, and no changes were observed [13].

Subsequently, the authors analyzed the expression of the proteins p53, p21^Cip1^^/Waf1^, cyclin-dependent kinase inhibitor 2A (p16^INK4a^), and the phosphorylation status of retinoblastoma (Rb) protein, through western blot analysis over 5 days. In *wt* HCT-116 cells treated with 10 μM of Uro-A and MPhA, a significant overexpression of p53 and p21^Cip1/Waf1^ was observed. On the other hand, there were no changes in p16^INK4a^/Rb pathway compared to the control cells, suggesting its inactivation. In treated Caco-2 cells (p53-null), no significant changes were observed in p53 expression or in the p16^INK4a^/Rb pathway, although a significant overexpression of p21^Cip1/Waf1^ was detected following treatment with 10 μM of Uro-A and MPhA. Moreover, in HT-29 treated cells (mutant p53), no alterations were observed in the expression of any of the markers [13].

Norden et al. (2019) treated both *wt* and p53-/- HCT-116 cells continuously with 20 μM of Uro-A for 25 days. The effects were evaluated using X-gal staining and cumulative population doublings (CPD). While p53-/- cells demonstrated increased cellular death and absence of SA-β-gal activity, *wt* cells exhibited complete cessation of proliferation, morphological changes consistent with senescent cells, becoming larger and flatter, and increased SA-β-gal activity [28].

#### 3.4.8 Induction of Autophagy

Zhao et al. (2018) performed a study in which they examined the morphological features of SW620 cells in autophagy after treatment with 1.5 or 15 μM of Uro-A. The transmission electron microscopy results detected the presence of autolysosomes, indicating the activation of autophagy. Furthermore, the conversion of green fluorescent protein-microtubule-associated protein 1 light chain 3 (GFP-LC3) was observed from a faint, diffused pattern to distinct fluorescent cytoplasmic puncta and an accumulation of GFP-LC3 after 24 h in a dose-independent manner. Moreover, western blot analysis demonstrated an increased conversion of the cytosolic form LC3-I into LC3-II, further supporting the induction of autophagy [26].

## 4 Discussion

This systematic review summarized the current scientific evidence regarding the anticancer potential of Uro-A in 5 well-established human CRC cell lines—Caco-2, HT-29, SW480, SW620 and HCT-116, as well as in one non-tumorigenic colon-derived cell line (CCD18-Co), based on 15 *in vitro* experimental studies. The results demonstrated that Uro-A exhibits multiple antitumor properties, including inhibition of cell proliferation and clonogenic growth, suppression of CSC-related properties, induction of cell cycle arrest and apoptosis, cell migration and invasion suppression, as well as induction of autophagy and cellular senescence.

### 4.1 Colorectal Cell Lines Heterogeneity and Susceptibility to Uro-A

It is well known that CRC cell lines constitute robust pre-clinical models as they represent different histological and molecular subtypes of CRC and preserve the main genetic mutations related to the primary tumor. Thus, this inherent variability among the cell lines enables the comparative evaluation of therapeutic approaches in a reproducible and controlled manner [33].

This is particularly relevant when considering the heterogeneous response to treatment with Uro-A, whose anticancer activity, although consistent in the multiple outcomes evaluated, differed according to the susceptibility profile of each cell line. For instance, the effects were greater in HCT-116 cells, which proved to be the most sensitive cells, followed by SW480, Caco-2 and HT-29 cell lines with intermediate sensitivity depending on the analytical experiment, and finally, SW620 and CCD18-Co cells, which exhibited greater resistance. Notably, this lower sensitivity can be explained by their intrinsic characteristics. SW620 cell line, derived from a metastasis, exhibits a more aggressive phenotype and enhanced migratory and invasive features, often associated with therapeutic resistance [34]. On the other hand, the resistance of CCD18-Co, a non-tumorigenic fibroblast cell line, suggests that Uro-A exhibits selective activity against cancer cells [13,29].

### 4.2 Dosage and Physiological Relevance

The response pattern was found to be related to the administered dose and incubation period. In the selected studies, Uro-A was tested at a wide range of concentrations (0.1–400 μM) and exposure times (24–72 h), with 50 and 100 μM for 24–48 h being the most frequently used and yielding the most consistent and significant effects. Overall, concentrations equal or below 25 μM required longer exposure or combination with other compounds to achieve relevant and statistically significant outcomes.

However, the physiological relevance of the *in vitro* concentrations used remains uncertain, as Uros undergo extensive Phase II metabolism [20]. This process converts Uros into hydrophilic molecules, more easily excreted by the organism, through conjugation reactions including glucuronidation, methylation, and sulfation [9,10,35].

It has been reported that Uros aglycones, particularly Uro-A, reach the highest levels-up to 100 μM—in the colonic lumen. Nevertheless, their conjugated counterparts, such as Uro-A and Uro-B glucuronides, are the primary circulating metabolites in humans, typically present at low μM concentrations [9,10,20]. Thus, although the *in vitro* concentrations used may be locally achievable in the colon following the intake of ET-rich products, their plasma levels are considerably lower [20], which limits the potential application of Uro-A as a systemic therapeutic agent.

### 4.3 Cell Proliferation, Metabolism and Drug Resistance

The findings of the selected studies systematically showed that Uro-A exerts an antiproliferative activity across all CRC cell lines. In general, the inhibitory effect was directly proportional to the dose and duration of treatment, except in HT-29 cells, which in some studies exhibited a time-independent response [20,21]. This behavior may be related to the methodological variability observed across different assays or to intrinsic biological characteristics of HT-29 cells, such as their ability to glucuronidate Uros through Phase II metabolism [12,20]. This biochemical process is mediated by uridine diphosphate glucuronosyltransferases (UGT), which catalyze the conjugation of glucuronic acid to the functional groups of a specific substrate [35]. According to previous reports, HT-29 cells exhibit hypomethylation in the promoter region of the uridine diphosphate glucuronosyltransferase family 1 member A1 *(UGT1A1)* gene [36], which leads to the overexpression of UGT1A isoforms, thereby enhancing their glucuronidation capacity [37]. These conjugated forms exhibit lower biological activity [25,35] and, consequently, reduced antiproliferative effects, which indicates that they may be used by cancer cells as a resistance mechanism to the Uros treatment [20,25].

This hypothesis is further supported by the studies performed by González-Sarrías et al. (2014, 2017), which revealed a marked increase of Uro-A glucuronides in HT-29 cells, a minimal presence in Caco-2 cells, and no detectable conjugates in SW480 cells following treatment with Uro-A aglycone [20,25]. These results confirm that HT-29 cells have a more active metabolism, characterized by a high glucuronidation rate, whereas Caco-2 and SW480 cells exhibit limited or absent metabolic activity [20]. Additionally, the same studies revealed that Caco-2, SW480, and HT-29 cells treated with Uro-A glucuronide exhibited decreased cell proliferation inhibition compared to the respective aglycone [20,25].

Besides Phase II metabolism, the decreased sensitivity of HT-29 cells to the effects of Uro-A may be related to adenosine triphosphate (ATP) binding cassette (ABC) transporters [20], which actively transport a wide range of substrates across the cell membrane through ATP hydrolysis. Therefore, these transporters have the ability to modulate the pharmacokinetic and pharmacological properties of their substrates, as well as mediate drug-drug interactions [38,39]. It is known that HT-29 cells have high expression of ABC transporters, including subfamily G2 (ABCG2) and multidrug resistance-related protein (MRP), in contrast with SW480 cells, which have lower expression [40,41]. Furthermore, several studies have described the role of ABC transporters in the transport of secondary plant metabolites, such as polyphenols [42], and have shown that Uro-A and its conjugates are substrates of these efflux pumps [38].

González-Sarrías et al. (2014) subsequently investigated the role of specific ABC transporter inhibitors in HT29 cells in order to verify their relationship with the cells’ susceptibility to Uros. The findings revealed that these inhibitors partially prevented the glucuronidation of Uro-A, making the cancer cells more sensitive to their antiproliferative effects. This suggests that ABC transporters, along with Phase II metabolism, contribute to a resistance mechanism in cancer cells [20].

In addition to Uro-A’s immediate antiproliferative effects, clonogenic assays also confirmed that this metabolite significantly compromises the long-term self-renewal of cancer cells by inhibiting colony formation and promoting irreversible structural changes, possibly through cellular DNA breakdown [13,15]. Since these assays mimic the features of malignant cells *in vivo* [43], these findings support the promising potential of Uro-A in cancer treatment.

### 4.4 Cellular and Molecular Mechanisms: Cell Cycle Arrest, Senescence, Apoptosis, and Autophagy

One of the key mechanisms underlying the inhibition of cellular proliferation is related to cell cycle arrest [44]. The cell cycle is an extremely regulated biological process that controls cell division and proliferation. Therefore, a defect in one of its regulatory proteins, such as cyclins, cyclin-dependent kinase (CDK) and cyclin-dependent kinase inhibitors (CKI), can lead to uncontrolled proliferation and, consequently, the development of diseases such as cancer [45]. CDKs are kinase proteins that can either be activated at specific checkpoints of the cell cycle by cyclins, allowing its progression, or neutralized by CKI, leading to cell cycle arrest. Unlike cyclins, whose protein levels oscillate throughout the cycle, CDKs remain stable, ensuring an orderly and controlled progression through the different phases [45].

In the selected studies, Uro-A consistently promoted a significant accumulation of cancer cells in the G_2_/M and S phases, in a dose- and time-dependent manner, accompanied by a consequent decrease in the cellular population in the G_0_/G_1_ phase. Some reports have shown that this effect is closely related to the modulation of the expression of regulatory proteins, key genes and signaling pathways essential for cell cycle progression [21,29,46].

**Figure 3:**
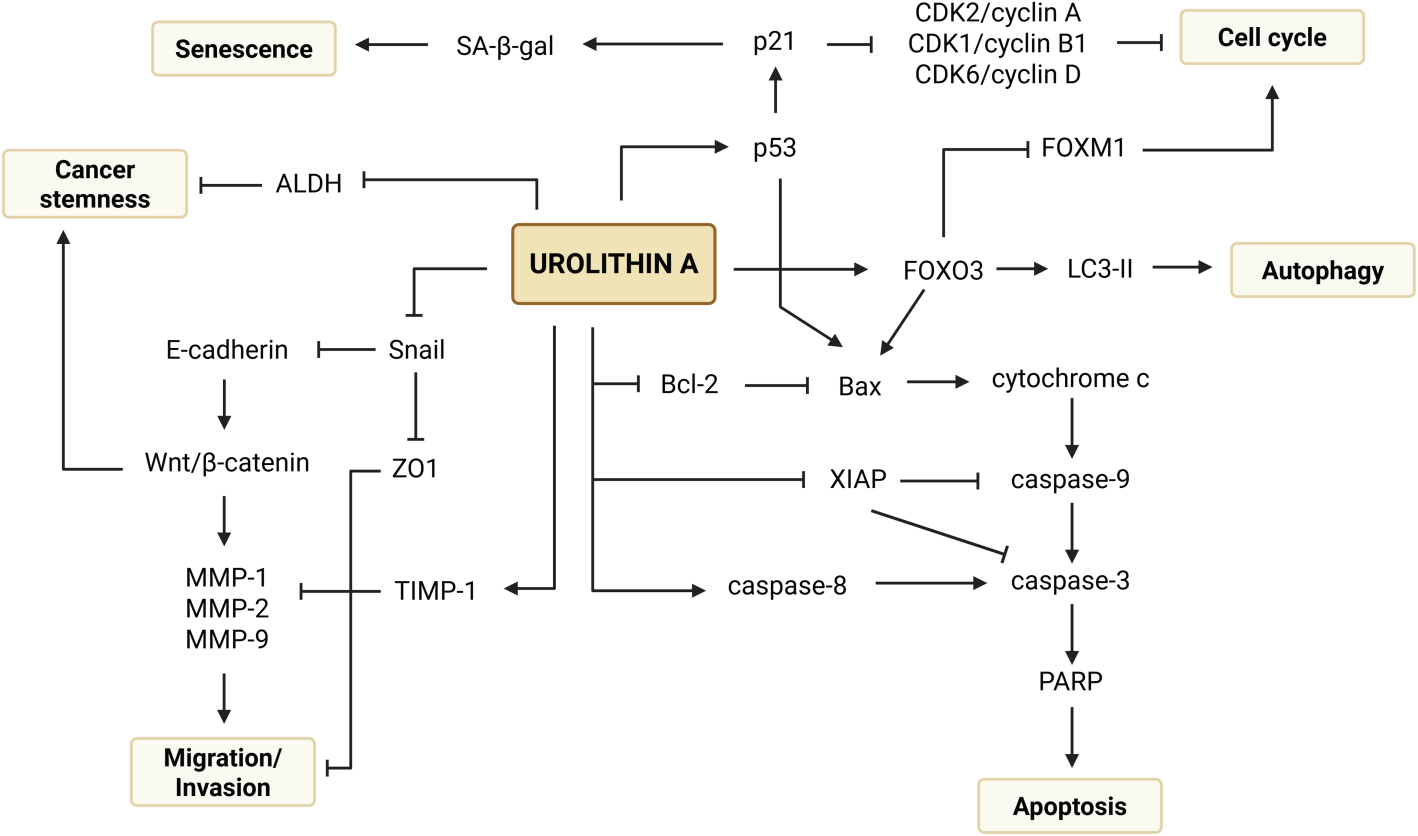
Mechanistic overview of the anticancer potential of urolithin A in colorectal cancer. Created with BioRender.com. Note: ALDH, aldehyde dehydrogenase; Bax, B-cell leukemia/lymphoma 2 associated X Protein; Bcl-2, B-cell leukemia/lymphoma 2 Protein; CDK1, cyclin-dependent kinase 1; CDK2, cyclin-dependent kinase 2; CDK6, cyclin-dependent kinase 6; FOXM1, forkhead box M1; FOXO3, forkhead box O3; LC3-II, microtubule-associated protein 1 light chain 3 II; MMP-1, metalloproteinase 1; MMP-2, metalloproteinase 2; MMP-9, metalloproteinase 9; p21, cyclin-dependent kinase inhibitor 1A; p53, tumor protein p53; PARP, poly (adenosine diphosphate-ribose) polymerase; SA-β-gal, senescence-associated β-galactosidase; Snail, zinc finger protein SNAI1; TIMP-1, inhibitor of metalloproteinases 1; XIAP, X-linked inhibitor of apoptosis protein; ZO1, zona occludens 1

In fact, cyclins A and B1, CDK6 and p21 were found to be increased in the studies conducted by Cho et al. (2015), González-Sarrías et al. (2015), and El-Wetidy et al. (2021) following treatment with Uro-A [21,22,29]. Cyclin A binds to CDK2 to drive S phase progression, while its association with CDK1 mediates the G_2_/M transition. In addition, the cyclin B1/CDK1 complex facilitates entry into M phase and Cyclin D/CDK6 regulates the G_0_/G_1_ phase [29,45]. On the other hand, p21 is a CKI that forms a stable complex with CDK1 and CDK2, preventing their interaction with cyclins and, consequently, the progression of the cell cycle in the S and G_2_/M phases, respectively [9,45]. Therefore, these molecular findings corroborate the observed cellular distribution patterns (Fig. 3) [21,22,29].

Notably, additional investigations have exhibited new insights into Uro-A’s anticancer mechanisms in CRC. A study by González-Sarrías et al. (2009) on Caco-2 cells treated with Uro-A showed an inhibition of cellular proliferation mediated by the upregulation of tumor suppressor genes (dual specificity phosphatase 6, *DUSP6*; and V-fos FBJ murine osteosarcoma viral oncogene homologue, *Fos*), and downregulation of growth factor receptors (fibroblast growth factor receptor 2, *FGFR2*) [47].

Furthermore, Sharma et al. (2010) demonstrated that Uro-A is an inhibitor of the Wnt/β-catenin signaling pathway, a highly critical pathway in the regulation of the cellular cycle that is strongly implicated in the pathogenesis of CRC when constitutively activated (Fig. 3) [48]. A study conducted by Norden et al. (2019) also revealed that the antiproliferative activity of Uro-A in *wt* HCT-116 cells is related to the modulation of p53 and tumor protein p53 induced glycolysis and apoptosis regulator (TIGAR) axis. This metabolite is capable of stabilizing p53, which is frequently mutated in CRC, and induces the expression of TIGAR, an inhibitor that reduces the glycolytic potential of cancer cells by decreasing the levels of fructose-2,6-bisphosphate [28].

Disruption of the cell cycle can also lead to cellular senescence, a post-mitotic and well-differentiated stage in which there is an irreversible and permanent arrest of cellular proliferation [49]. Giménez-Bastida et al. (2020) studied the underlying mechanisms of cell cycle arrest and anti-clonogenic growth and observed an increase in the activity of the enzyme SA-β-gal, a marker found in senescent cells, exclusively in HCT-116 cells (*wt* p53) treated with Uro-A. Subsequently, the results revealed that Uro-A induces p53-dependent cellular senescence due to the overexpression of p53 and p21^Cip1/Waf1^ [13] (Fig. 3). This finding is corroborated by Norden et al. (2019), who proved that the induction of senescence-like growth arrest by Uro-A is dependent on a functional p53 (*wt* cells), as cells HCT-116 p53-/- or p21-/- did not show signs of senescence after treatment [28].

Besides cell cycle arrest, the results of the studies included in this systematic review provided solid evidence that Uro-A can inhibit cell proliferation by inducing programmed cell death, as its treatment led to a significant increase in the percentage of early and late apoptotic cancer cells.

As the name suggests, early apoptotic cells are in the initial stage of apoptosis and exhibit characteristic morphological changes such as pyknosis and cell shrinkage, while still maintaining cellular membrane integrity. In contrast, late apoptotic cells are involved in the more advanced stages of the process and display irreversible features including cellular membrane blebbing, karyorrhexis, and the formation of apoptotic bodies. Distinguishing between these two types of cells is essential, as several features of apoptosis can be easily mistaken for necrosis [50,51].

Moreover, it was shown that the induction of apoptosis by Uro-A occurred through the modulation of both intrinsic and extrinsic pathways. Activation of the intrinsic (mitochondrial) pathway was confirmed by mitochondrial membrane potential depolarization, overexpression of cytochrome c, cleaved caspase-9 (active form) and the pro-apoptotic protein Bax, as well as depletion of the anti-apoptotic proteins Bcl-2 and XIAP. Meanwhile, the activation of the extrinsic (death receptor-initiated) pathway was supported by increased levels of caspase-8. The convergence of these pathways was sustained by an overexpression of cleaved caspase-3 and PARP, indicating the activation of the final execution pathway of apoptosis. Additional findings have shown that the induction of these apoptotic pathways might be under the influence of p53 and p21, as they were found to be overexpressed in some studies (Fig. 3) [21,22,29,30].

Although the induction of apoptosis occurred generally in a concentration-dependent manner, there is evidence that, at lower concentrations, Uro-A can alternatively exert an anticancer activity by activating the autophagic pathways [26]. This was demonstrated in a study by Zhao et al. (2018), in which SW620 cells exposed to lower concentrations of Uro-A than those used to evaluate apoptosis exhibited morphological and molecular features suggestive of autophagy. These included the presence of autolysosomes – cytoplasm vacuoles with cellular fragments and organelles –, the appearance of a cytoplasmic puncta pattern of the GFP-LC3 fusion protein, indicating the translocation of LC3 to the membrane of the autophagosomes [52], and the conversion of the cytosolic form LC3-I to the autophagic form LC3-II, confirming the induction of the autophagic process (Fig. 3) [26,53]. In addition to Uro-A, there is robust evidence that other secondary plant metabolites, including phenolic, terpenoid, and alkaloid derivatives, also displayed an autophagy-inducing potential in CRC cell lines [53].

### 4.5 Inhibition of Migration, Invasion, and Effects on Cancer Stem Cells

Metastasis is a group of consecutive biological events that predict the severity of a tumor, being the main cause of mortality in cancer patients [54]. Along with its impact on cellular viability, Uro-A has also been shown to suppress the migration and invasion of cancer cells, including those derived from metastatic cancer (SW620) and resistant to conventional treatments (HCT-116-FUR and SW480-FUR) [15,26,30]. These findings were corroborated by a delay and decrease in the number of migratory cells, reduced expression of matrix-degrading endopeptidases (MMP-1, MMP-2, and MMP-9), as well as transcription factors and mediators of EMT (Snail protein and β-catenin). Simultaneously, an increase in the expression of proteins involved in cellular adhesion (E-cadherin), tight junctions (ZO1), and inhibitors of MMP activity (TIMP-1) were observed. Thus, by modulating key proteins, Uro-A emerges as a promising agent in preventing tumor progression (Fig. 3) [15,26,30,55].

CSCs are cells with strong implications in cancer pathogenesis and metastasis, as well as in resistance to chemotherapy treatments, standing out for their capacity for self-renewal, pluripotency, and metabolic reprogramming. Given their importance, the use of three-dimensional models that simulate *in vivo* behavior, such as colonospheres, allows for the assessment of their fundamental properties and the development of more effective therapeutic strategies [24,32,56]. In this context, two mixtures of ET derivatives, including Uro-A, were tested to assess their potential anticancer effects on these cells. A decrease in the number and size of colonospheres, accompanied by a reduction in the subpopulation with ALDH^high^ activity, a well-established marker of SC, was detected. ALDH is an enzyme that catalyzes the oxidation of aldehydes into carboxylic acids, playing a detoxifying role. Moreover, it is involved in the synthesis of retinoic acid and in biological processes, including cell proliferation and differentiation, carcinogenesis, stemness and therapeutic resistance (Fig. 3) [24,57,58]. Additionally, possible changes in the expression of CD133 were examined. CD133 is a transmembrane glycoprotein that has been identified as a SC surface marker involved in cell metabolism, differentiation, stemness, and tumorigenesis [24,59]. However, the treatment had no effect, indicating the existence of different CSC subpopulations and reflecting the need for targeted therapies [24,32,56].

### 4.6 Dietary Context and Combination Therapies

The pathogenesis of CRC, recognized as a multifactorial disease, results from the interaction between genetic predisposition, lifestyle-related habits and environmental factors. Currently, there is robust scientific evidence that diet, as a modifiable risk factor, plays a crucial role in the incidence of this disease. Among the various dietary patterns, those characterized by high consumption of fibers, fruits, and vegetables, and low intake of processed foods and fats, are associated with a lower risk of developing CRC [60–62]. This protective effect is also directly associated with gut microbiota, particularly due to the fermentation of compounds such as dietary fibers. One of the main products of this metabolism is SB, a short-chain fatty acid that has various anticancer properties, such as inhibiting cellular proliferation, modulating inflammatory processes, maintaining the integrity of the colonic epithelium, and activating apoptosis [62].

In this context, although Uro-A shows promising effects when administered alone, there is evidence that its combination with other natural compounds, such as SB, increases its efficacy compared to monotherapy. Indeed, this additive interaction manifested as greater inhibition of cellular growth, induction of apoptosis, and modulation of the inflammatory tumor microenvironment, with decreased expression of the inflammatory enzymes inducible nitric oxide synthase (iNOS) and cyclooxygenase-2 (COX2) [27].

The administration of combined therapies becomes even more relevant when considering the interindividual variability among CRC patients. Several studies have studied the response of cancer cells treated with MPhA and MPhB and have demonstrated equally promising anticancer potential in almost all the evaluated outcomes. Overall, the anticancer activity did not vary extensively between the two mixtures, although the one with higher concentrations of Uro-A appeared to induce slightly higher inhibition in tumor cells. Thus, the administration of mixtures that qualitatively and quantitatively mimic the different human metabolic profiles allows for the reproduction of physiological characteristics in *in vivo* contexts [13,23,24].

Individual heterogeneity, along with the side effects of chemotherapy and chemoresistance, has further increased the need to identify alternative strategies capable of overcoming these obstacles and enhancing the efficacy of conventional treatments [30]. In fact, the results proved that the combination of Uro-A with 5-FU, 5^′^-DFUR, or oxaliplatin improved the therapeutic effect of these chemotherapeutic agents through an additive or synergistic interactions, supporting the role of this metabolite as an adjuvant in cancer treatment. The magnitude of the response depended on both the cell line and genotype. Among the tested models, SW480 and HCT-116 (*wt*) cells were the most responsive, whereas p53-/- cells exhibited increased resistance, particularly to the combination of Uro-A and oxaliplatin. Additionally, the effects varied according to the combination type, with Uro-A + 5-FU exhibiting notably stronger cytotoxic effects compared to Uro-A + 5^′^-DFUR.

Taken together, these co-treatments increased chemosensitivity of cancer cells and their respective resistant variants while also amplified direct antitumor effects, such as inhibition of cell proliferation, cell cycle arrest, induction of apoptosis, and suppression of migratory and invasive features [22,28,30]. According to Ghosh et al. (2022), Uro-A acts as a chemosensitizer in FUR variants by negatively regulating the expression of the transporters responsible for removing intracellular 5-FU. This occurs through the modulation of the forkhead box O3/forkhead box M1 (FOXO3/FOXM1) axis by increasing and decreasing the expression of the transcription factors FOXO3 and FOXM1, respectively, which leads to an increase in the cellular concentration of the chemotherapeutic agent (Fig. 3) [30].

### 4.7 Uro-A’s Role in Other Malignancies

Although this review focused on CRC, the anticancer mechanisms of Uro-A extend to a wide variety of other malignancies. For instance, preclinical studies have reported its effects in hepatobiliary and genitourinary tumors, as well as in hormone-dependent cancers, including uterine, breast and prostate [7,8,63].

Similarly to what occurs in CRC, Uro-A has demonstrated the ability to inhibit cell proliferation through cell cycle arrest in S and G2/M phases, activate apoptotic pathways, and induce cellular senescence in *in vitro* models, in a dose- and time-dependent manner. In addition, this metabolite also suppressed the migratory and invasive features of the cancer cell lines. As expected, these effects were associated to its capacity to modulate gene expression through downregulation of oncogenes, upregulation of tumor suppressors and inhibition of survival pathways [7,8,63].

Regarding hormone-sensitive tissues, Uro-A has been shown to regulate receptor signaling and hormone-related pathways, including the activation of estrogen receptor signaling in the endometrium, aromatase-inhibitory and antiestrogenic effects in breast tissue, and downregulation of androgen receptor activity and secretory markers in the prostate [8].

Despite promising results, several authors have highlighted the challenges of using Uro-A as a potential therapeutic agent in systemic tissues. This arises from Uros gut metabolism by microbiota, which inevitably leads to higher local concentrations of its aglycone forms. As previously noted, Uros circulating systemically are predominantly in their conjugated form, resulting in reduced anticancer activity in these tissues [7,8].

## 5 Study and Evidence Limitations

This systematic review has some limitations that deserve discussion. The use of only three databases, as well as the search strategy employed and the defined eligibility criteria, may have led to the omission of studies relevant to the topic. The absence of well-established automatic and validated tools for assessing the risk of bias in *in vitro* assays required a manual evaluation by the authors, which may have compromised the assessment of the methodological quality of the studies. Furthermore, the lack of standardization in the tested concentration ranges and exposure times, as well as the variability of laboratory methods used to assess different outcomes, hinders comparative analysis across studies.

Despite providing valuable insights, *in vitro* studies present inherent biological limitations that deserve to be addressed. These include the inability of cell models to accurately reproduce the tissue architecture of the tumor, its surrounding microenvironment, and the complex interactions with the extracellular matrix. Consequently, the results may either underestimate or overestimate the efficacy of the compounds under investigation due to lack of systemic factors, such as immune interactions, drug metabolism and bioavailability [64,65], which hinders the translation to clinical practice. Moreover, the over-reliance on specific cell lines, such as HT-29 and SW480, limits the extrapolation of the findings, as these lines do not fully cover the genetic and phenotypic heterogeneity of the different histological subtypes of CRC.

Additionally, the physicochemical properties of Uro-A, including poor water solubility, local delivery, microbiota- and metabolic phenotype-dependent biotransformation, low oral absorption, extensive Phase II metabolism, and pharmacokinetic variability, may compromise its efficacy and challenge the clinical interpretation of *in vitro* data [63].

To overcome these limitations, it is crucial to promote future research on dietary strategies in the context of CRC. Such efforts may benefit from incorporating patient-derived organoids/spheroids, patient-derived xenografts or clinical trials, as well as advanced approaches such as nanotechnology-based delivery systems and gut microbiota modulation [63,64].

## 6 Conclusion

This systematic review synthesized the current scientific evidence regarding the anticancer potential of Uro-A in human CRC cell lines. Through the analysis of the included studies, it was possible to demonstrate that Uro-A exerts significant and consistent anticancer effects, including inhibition of cell proliferation and clonogenic growth, induction of apoptosis, autophagy, cellular senescence, cell cycle arrest, as well as suppression of cell migration, invasion, and CSC-related properties.

Although the effects of Uro-A were generally consistent across various outcomes, its efficacy was shown to depend on the administered concentration and duration of exposure, with concentrations between 50 and 100 μM and incubation periods of 24–48 h yielding the most consistent and statistically significant results. While such concentrations may be locally achievable in the colon following the consumption of ET-rich products, plasmatic levels observed *in vivo* are typically much lower and mostly present as Uro-A glucuronides, a less biologically active form.

The observed variability in response to Uro-A appears to be closely related to distinct susceptibility profiles to the compound due to the intrinsic characteristics of each cell line, including cellular origin and phenotype, Phase II metabolic profile, and overexpression of efflux transporters.

Moreover, the antitumor effects of Uro-A were found to be associated with the modulation of critical signaling pathways involved in carcinogenesis, as well as with alterations in the expression of key regulatory genes and proteins essential to tumor progression.

Uro-A’s selectivity towards tumor cells, along with additive and synergistic interactions observed when co-administered with natural compounds (SB and ET derivatives), and conventional chemotherapeutic agents (5-FU, 5^′^-DFUR and oxaliplatin), supports its potential as an adjuvant in cancer treatment.

Despite the promising findings, the studies included in this review exhibited relevant methodological limitations, particularly the lack of standardization in the concentrations tested, exposure durations, and laboratory methods employed. Additionally, it is important to highlight the preclinical and preliminary nature of the evidence presented, which hinders the generalization of results and limits their direct extrapolation to *in vivo* contexts or clinical practice. Given the limited clinical evidence in humans supporting the full efficacy of Uro-A in the context of CRC, it is essential to develop future studies to validate the *in vitro* results and determine its true therapeutic, safety and preventive potential.

By identifying these gaps in the current body of knowledge, this review aims to contribute to the establishment of a more solid scientific foundation that may help guide future clinical investigations, thereby supporting the development of more effective dietary and pharmacological strategies for CRC management, such as approaches focused on bioavailability and delivery enhancement of Uro-A or personalized interventions based on gut metabotypes.

## Supplementary Materials





## Data Availability

Not applicable.

## Supplementary Materials

The supplementary material is available online at https://www.techscience.com/doi/10.32604/or.2025.070276/s1.

## Abbreviations

5^′^-DFUR: 5^′^-Deoxy-5-fluorouridine
5-FU: 5-Fluorouracil
ABC: Adenosine triphosphate binding cassette
ABCG2: Adenosine triphosphate binding cassette subfamily G2
ALDH: Aldehyde dehydrogenase
Annexin V/PI: Annexin V/propidium iodide
Annexin V-FITC/PI: Annexin V-fluorescein isothiocyanate/propidium iodide
ATP: Adenosine triphosphate
Bax: B-cell leukemia/lymphoma 2 associated X Protein
Bcl-2: B-cell leukemia/lymphoma 2 Protein
C_1_: Low concentration
C_2_: High concentration
CD133: Prominin-1
CDK: Cyclin-dependent kinase
CDK1: Cyclin-dependent kinase 1
CDK2: Cyclin-dependent kinase 2
CDK6: Cyclin-dependent kinase 6
CI: Combination index
CKI: Cyclin-dependent kinase inhibitors
COX2: Cyclooxygenase-2
CPD: Cumulative population doublings
CRC: Colorectal cancer
CSC: Cancer stem cells
*DUSP6*: Dual specificity phosphatase 6
EA: Ellagic acid
EMT: Epithelial-mesenchymal transition
ET: Ellagitannins
FDG: 5-dodecanoylaminofluorescein di-β-D-galactopyranoside
*FGFR2*: Fibroblast growth factor receptor 2
FUR: 5-Fluorouracil chemoresistant
*Fos*: V-fos FBJ murine osteosarcoma viral oncogene homologue
FOXM1: Forkhead box M1
FOXO3: Forkhead box O3
GFP-LC3: Green fluorescent protein-microtubule-associated protein 1 light chain 3
h: Hours
HHDP: Hexahydroxydiphenoyl
IC_50_: Half-maximal inhibitory concentration
IsoUro-A: Isourolithin A
iNOS: Inducible nitric oxide synthase
JC-1: 5,5^′^,6,6^′^-tetrachloro-1,1^′^,3,3^′^-tetraethyl-benzimidazolocarbocyanine iodide
Ki-67: Marker of proliferation
MeSH: Medical Subject Headings
MMP: Metalloproteinase 1
MMP: Metalloproteinase 2
MMP: Metalloproteinase 9
MPhA: Mixtures of ET metabolites mimicking metabotype A
MPhB: Mixtures of ET metabolites mimicking metabotype B
MRP: Multidrug resistance-related protein
MTS: 3-(4,5-dimethylthiazol-2-yl)-5-(3-carboxymethoxyphenyl)-2-(4-sulfophenyl)-2H-tetrazolium
MTT: 3-(4,5-dimethylthiazol-2-yl)-2,5-diphenyltetrazolium bromide
p16: Cyclin-dependent kinase inhibitor 2A
p21: Cyclin-dependent kinase inhibitor 1A
p53: Tumor protein p53
PARP: Poly (adenosine diphosphate-ribose) polymerase
PI: Propidium iodide
PICO: Patient/population, intervention, comparison, and outcome
PCNA: Proliferating cell nuclear antigen
PRISMA: Preferred reporting items for systematic reviews and meta-analyses
PROSPERO: International prospective register of systematic reviews
Ref: Reference
SA-β-gal: Senescence-associated β-galactosidase
SB: Sodium butyrate
SC: Stem cells
Snail: Zinc finger protein SNAI1
TIGAR: Tumor protein p53 induced glycolysis and apoptosis regulator
TIMP-1: Inhibitor of metalloproteinases 1
ToxRTool: Toxicological data reliability assessment tool
UGT: Uridine diphosphate glucuronosyltransferases
*UGT1A1*: Uridine diphosphate glucuronosyltransferase family 1 member A1
Uro: Urolithin
Uro-A: Urolithin A
Uro-AR: Urolithin AR
Uro-B: Urolithin B
Uro-C: Urolithin C
Uro-CR: Urolithin CR
Uro-D: Urolithin D
Uro-E: Urolithin E
Uro-M5: Urolithin M5
Uro-M6: Urolithin M6
Uro-M6R: Urolithin M6R
Uro-M7: Urolithin M7
Uro-M7R: Urolithin M7R
*wt*: wild type
X-gal: 5-bromo-4-chloro-3- indolyl-B-d-galactopyranoside
XIAP: X-linked inhibitor of apoptosis protein
ZO1: Zona occludens 1

## References

[1] Bray F, Laversanne M, Sung H, Ferlay J, Siegel RL, Soerjomataram I, et al. Global cancer statistics 2022: GLOBOCAN estimates of incidence and mortality worldwide for 36 cancers in 185 countries. CA A Cancer J Clin. 2024;74(3):229–63. doi:10.3322/caac.21834; 38572751

[2] Matsuda T, Fujimoto A, Igarashi Y. Colorectal cancer: epidemiology, risk factors, and public health strategies. Digestion. 2025;106(2):91–9. doi:10.1159/000543921; 39938491

[3] World Health Organization. Colorectal Cancer [Internet]. 2023 [cited 2025 Jun 21]. Available from: https://www.who.int/news-room/fact-sheets/detail/colorectal-cancer.

[4] Haynes J, Manogaran P. Mechanisms and strategies to overcome drug resistance in colorectal cancer. Int J Mol Sci. 2025;26(5):1988. doi:10.3390/ijms26051988; 40076613 PMC11901061

[5] AlAli M, Alqubaisy M, Aljaafari MN, AlAli AO, Baqais L, Molouki A, et al. Nutraceuticals: transformation of conventional foods into health promoters/disease preventers and safety considerations. Molecules. 2021;26(9):2540. doi:10.3390/molecules26092540; 33925346 PMC8123587

[6] Divella R, Daniele A, Savino E, Paradiso A. Anticancer effects of nutraceuticals in the Mediterranean diet: an epigenetic diet model. Cancer Genom Proteom. 2020;17(4):335–50. doi:10.21873/cgp.20193; 32576579 PMC7367609

[7] García-Villalba R, Giménez-Bastida JA, Cortés-Martín A, Ávila-Gálvez MÁ, Tomás-Barberán FA, Selma MV, et al. Urolithins: a comprehensive update on their metabolism, bioactivity, and associated gut microbiota. Mol Nutr Food Res. 2022;66(21):2101019. doi:10.1002/mnfr.202101019; 35118817 PMC9787965

[8] Al-Harbi SA, Abdulrahman AO, Zamzami MA, Khan MI. Urolithins: the gut based polyphenol metabolites of ellagitannins in cancer prevention, a review. Front Nutr. 2021;8:647582. doi:10.3389/fnut.2021.647582; 34164422 PMC8215145

[9] Liberal J. Fragaria vesca leaf as a source of bioactive phytochemicals—a focus on ellagitannins and their human microflora metabolites [dissertation]. Coimbra, Portugal: Universidade de Coimbra; 2015.

[10] Raimundo AF, Ferreira S, Tomás-Barberán FA, Santos CN, Menezes R. Urolithins: diet-derived bioavailable metabolites to tackle diabetes. Nutrients. 2021;13(12):4285. doi:10.3390/nu13124285; 34959837 PMC8705976

[11] Djedjibegovic J, Marjanovic A, Panieri E, Saso L. Ellagic acid-derived urolithins as modulators of oxidative stress. Oxid Med Cell Longev. 2020;2020(1):5194508. doi:10.1155/2020/5194508; 32774676 PMC7407063

[12] Lin IC, Wu JY, Fang CY, Wang SC, Liu YW, Ho ST. Absorption and metabolism of urolithin a and ellagic acid in mice and their cytotoxicity in human colorectal cancer cells. Evid Based Complement Alternat Med. 2023;2023(1):8264716. doi:10.1155/2023/8264716; 37706115 PMC10497365

[13] Giménez-Bastida JA, Ávila-Gálvez MÁ, Espín JC, González-Sarrías A. The gut microbiota metabolite urolithin A, but not other relevant urolithins, induces p53-dependent cellular senescence in human colon cancer cells. Food Chem Toxicol. 2020;139:111260. doi:10.1016/j.fct.2020.111260; 32179165

[14] Tow WK, Chee PY, Sundralingam U, Palanisamy UD. The therapeutic relevance of urolithins, intestinal metabolites of ellagitannin-rich food: a systematic review of *in vivo* studies. Nutrients. 2022;14(17):3494. doi:10.3390/nu14173494; 36079752 PMC9460125

[15] El-Wetidy MS, Rady MI, Rady I, Helal H. Urolithin A affects cellular migration and modulates matrix metalloproteinase expression in colorectal cancer cells. Cell Biochem Funct. 2024;42(3):e4019. doi:10.1002/cbf.4019; 38622949

[16] Page MJ, McKenzie JE, Bossuyt PM, Boutron I, Hoffmann TC, Mulrow CD, et al. The PRISMA, 2020 statement: an updated guideline for reporting systematic reviews. BMJ. 2021;18(3):e1003583. doi:10.1136/bmj.n71; 33780438 PMC8007028

[17] PROSPERO. Unveiling the anticancer potential of urolithin A in colorectal cancer: a systematic review [Internet]. 2025 [cited 2025 July 1]. Available from: https://www.crd.york.ac.uk/PROSPERO/view/CRD420251070874.

[18] Joint Research Centre EC. ToxRTool—toxicological data reliability assessment tool [Internet]. 2025 [cited 2025 Jul 1]. Available from: https://joint-research-centre.ec.europa.eu/scientific-tools-and-databases-0/toxrtool-toxicological-data-reliability-assessment-tool_en.

[19] American Type Culture Collection. American type culture collection (ATCC) [Internet]. 2025 [cited 2025 Jul 1]. Available from: https://www.atcc.org/.

[20] González-Sarrías A, Giménez-Bastida JA, Núñez-Sánchez MÁ, Larrosa M, García-Conesa MT, Tomás-Barberán FA, et al. Phase-II metabolism limits the antiproliferative activity of urolithins in human colon cancer cells. Eur J Nutr. 2014;53(3):853–64. doi:10.1007/s00394-013-0589-4; 24077694

[21] Cho H, Jung H, Lee H, Yi HC, Kwak HK, Hwang KT. Chemopreventive activity of ellagitannins and their derivatives from black raspberry seeds on HT-29 colon cancer cells. Food Funct. 2015;6(5):1675–83. doi:10.1039/c5fo00274e; 25906041

[22] González-Sarrías A, Tomé-Carneiro J, Bellesia A, Tomás-Barberán FA, Espín JC. The ellagic acid-derived gut microbiota metabolite, urolithin A, potentiates the anticancer effects of 5-fluorouracil chemotherapy on human colon cancer cells. Food Funct. 2015;6(5):1460–9. doi:10.1039/c5fo00120j; 25857357

[23] González-Sarrías A, Núñez-Sánchez MÁ, Tomé-Carneiro J, Tomás-Barberán FA, García-Conesa MT, Espín JC. Comprehensive characterization of the effects of ellagic acid and urolithins on colorectal cancer and key-associated molecular hallmarks: microRNA cell specific induction of CDKN1A (p21) as a common mechanism involved. Mol Nutr Food Res. 2016;60(4):701–16. doi:10.1002/mnfr.201500780; 26634414

[24] Núñez-Sánchez MÁ, Karmokar A, González-Sarrías A, García-Villalba R, Tomás-Barberán FA, García-Conesa MT, et al. *In vivo* relevant mixed urolithins and ellagic acid inhibit phenotypic and molecular colon cancer stem cell features: a new potentiality for ellagitannin metabolites against cancer. Food Chem Toxicol. 2016;92:8–16. doi:10.1016/j.fct.2016.03.011; 26995228

[25] González-Sarrías A, Núñez-Sánchez MÁ, García-Villalba R, Tomás-Barberán FA, Espín JC. Antiproliferative activity of the ellagic acid-derived gut microbiota isourolithin A and comparison with its urolithin A isomer: the role of cell metabolism. Eur J Nutr. 2017;56(2):831–41. doi:10.1007/s00394-015-1131-7; 26680596

[26] Zhao W, Shi F, Guo Z, Zhao J, Song X, Yang H. Metabolite of ellagitannins, urolithin A induces autophagy and inhibits metastasis in human sw620 colorectal cancer cells. Mol Carcinog. 2018;57(2):193–200. doi:10.1002/mc.22746; 28976622 PMC5814919

[27] Tortora K, Femia AP, Romagnoli A, Sineo I, Khatib M, Mulinacci N, et al. Pomegranate by-products in colorectal cancer chemoprevention: effects in apc-mutated pirc rats and mechanistic studies *in vitro* and *ex vivo*. Mol Nutr Food Res. 2018;62(2):1–10. doi:10.1002/mnfr.201700401. 28948694

[28] Norden E, Heiss EH. Urolithin A gains in antiproliferative capacity by reducing the glycolytic potential via the p53/TIGAR axis in colon cancer cells. Carcinogenesis. 2019;40(1):93–101. doi:10.1093/carcin/bgy158; 30418550 PMC6412115

[29] El-Wetidy MS, Ahmad R, Rady I, Helal H, Rady MI, Vaali-Mohammed MA, et al. Urolithin A induces cell cycle arrest and apoptosis by inhibiting Bcl-2, increasing p53-p21 proteins and reactive oxygen species production in colorectal cancer cells. Cell Stress Chaperones. 2021;26(3):473–93. doi:10.1007/s12192-020-01189-8; 33666815 PMC8065090

[30] Ghosh S, Singh R, Vanwinkle ZM, Guo H, Vemula PK, Goel A, et al. Microbial metabolite restricts 5-fluorouracil-resistant colonic tumor progression by sensitizing drug transporters via regulation of FOXO3-FOXM1 axis. Theranostics. 2022;12(12):5574–95. doi:10.7150/thno.70754; 35910798 PMC9330515

[31] Tiwari A, Tiwari V, Verma S, Sharma A. Network pharmacology, molecular docking, simulation studies of urolithin A by inhibiting AKT and caspase pathways against colorectal cancer. J Biol Regul Homeost Agents. 2024;38(6):5175–94. doi:10.23812/j.biol.regul.homeost.agents.20243806.414.

[32] Shaheen S, Ahmed M, Lorenzi F, Nateri AS. Spheroid-formation (colonosphere) assay for *in vitro* assessment and expansion of stem cells in colon cancer. Stem Cell Rev Rep. 2016;12(4):492–9. doi:10.1007/s12015-016-9664-6; 27207017 PMC4919387

[33] Mooi JK, Luk IY, Mariadason JM. Cell line models of molecular subtypes of colorectal cancer. Methods Mol Biol. 2018;1765:3–26. doi:10.1007/978-1-4939-7765-9_1; 29589298

[34] Luo F, Li J, Wu S, Wu X, Chen M, Zhong X, et al. Comparative profiling between primary colorectal carcinomas and metastases identifies heterogeneity on drug resistance. Oncotarget. 2016;7(39):63937–49. doi:10.18632/oncotarget.11570; 27613840 PMC5325415

[35] Rowland A, Miners JO, MacKenzie PI. The UDP-glucuronosyltransferases: their role in drug metabolism and detoxification. Int J Biochem Cell Biol. 2013;45(6):1121–32. doi:10.1016/j.biocel.2013.02.019; 23500526

[36] Gagnon JF, Bernard O, Villeneuve L, Têtu B, Guillemette C. Irinotecan inactivation is modulated by epigenetic silencing of UGT1A1 in colon cancer. Clin Cancer Res. 2006;12(6):1850–8. doi:10.1158/1078-0432.CCR-05-2130; 16551870

[37] Liu M, Wang Q, Liu F, Cheng X, Wu X, Wang H, et al. UDP-glucuronosyltransferase 1A compromises intracellular accumulation and anti-cancer effect of tanshinone IIA in human colon cancer cells. PLoS One. 2013;8(11):e79172. doi:10.1371/journal.pone.0079172; 24244442 PMC3828323

[38] González-Sarrías A, Miguel V, Merino G, Lucas R, Morales JC, Tomás-Barberán F, et al. The gut microbiota ellagic acid-derived metabolite urolithin A and its sulfate conjugate are substrates for the drug efflux transporter breast cancer resistance protein (ABCG2/BCRP). J Agric Food Chem. 2013;61(18):4352–9. doi:10.1021/jf4007505; 23586460

[39] Rees DC, Johnson E, Lewinson O. ABC transporters: the power to change. Nat Rev Mol Cell Biol. 2009;10(3):218–27. doi:10.1038/nrm2646; 19234479 PMC2830722

[40] Kim JH, Park JM, Roh YJ, Kim IW, Hasan T, Choi MG. Enhanced efficacy of photodynamic therapy by inhibiting ABCG2 in colon cancers. BMC Cancer. 2015;15:504. doi:10.1186/s12885-015-1514-4; 26149077 PMC4494642

[41] Park JW, Jung KH, Byun Y, Lee JH, Moon SH, Cho YS, et al. ATP-binding cassette transporters substantially reduce estimates of ALDH-positive cancer cells based on aldefluor and AldeRed588 assays. Sci Rep. 2019;9(1):6462. doi:10.1038/s41598-019-42954-9; 31015586 PMC6478741

[42] Yazaki K. ABC transporters involved in the transport of plant secondary metabolites. FEBS Lett. 2006;580(4):1183–91. doi:10.1016/j.febslet.2005.12.009; 16364309

[43] Franken NAP, Rodermond HM, Stap J, Haveman J, van Bree C. Clonogenic assay of cells *in vitro*. Nat Protoc. 2006;1(5):2315–9. doi:10.1038/nprot.2006.339; 17406473

[44] Tomás-Barberán FA, González-Sarrías A, García-Villalba R, Núñez-Sánchez MA, Selma MV, García-Conesa MT, et al. Urolithins, the rescue of “old” metabolites to understand a “new” concept: metabotypes as a nexus among phenolic metabolism, microbiota dysbiosis, and host health status. Mol Nutr Food Res. 2017;61(1):1500901. doi:10.1002/mnfr.201500901. 27158799

[45] Vermeulen K, Van Bockstaele DR, Berneman ZN. The cell cycle: a review of regulation, deregulation and therapeutic targets in cancer. Cell Prolif. 2003;36(3):131–49. doi:10.1046/j.1365-2184.2003.00266.x; 12814430 PMC6496723

[46] Muku GE, Murray IA, Espín JC, Perdew GH. Urolithin a is a dietary microbiota-derived human aryl hydrocarbon receptor antagonist. Metabolites. 2018;8(4):86. doi:10.3390/metabo8040086; 30501068 PMC6315438

[47] González-Sarrías A, Espín JC, Tomás-Barberán FA, García-Conesa MT. Gene expression, cell cycle arrest and MAPK signalling regulation in Caco‐2 cells exposed to ellagic acid and its metabolites, urolithins. Mol Nutr Food Res. 2009;53(6):686–98. doi:10.1002/mnfr.200800150; 19437480

[48] Sharma M, Li L, Celver J, Killian C, Kovoor A, Seeram NP. Effects of fruit ellagitannin extracts, ellagic acid, and their colonic metabolite, urolithin A, on WNT signaling. J Agric Food Chem. 2010;58(7):3965–9. doi:10.1021/jf902857v; 20014760 PMC2850963

[49] Tominaga K. The emerging role of senescent cells in tissue homeostasis and pathophysiology. Pathobiol Aging Age Relat Dis. 2015;5:27743. doi:10.3402/pba.v5.27743; 25994420 PMC4439419

[50] Elmore S. Apoptosis: a review of programmed cell death. Toxicol Pathol. 2007;35(4):495–516. doi:10.1080/01926230701320337; 17562483 PMC2117903

[51] Brauchle E, Thude S, Brucker SY, Schenke-Layland K. Cell death stages in single apoptotic and necrotic cells monitored by Raman microspectroscopy. Sci Rep. 2014;4:4698. doi:10.1038/srep04698; 24732136 PMC3986703

[52] Zhang L, Zhao J, Ding WX, Xia M. GFP-LC3 high-content assay for screening autophagy modulators. In: High-throughput screening assays in toxicology. New York, NY, USA: Springer; 2022. p. 83–9. doi:10.1007/978-1-0716-2213-1_9. PMC9438799 35294758

[53] Zhang B, Liu L. Autophagy is a double-edged sword in the therapy of colorectal cancer (review). Oncol Lett. 2021;21(5):378. doi:10.3892/ol.2021.12639; 33777202 PMC7988732

[54] Gerstberger S, Jiang Q, Ganesh K. Metastasis. Cell. 2023;186(8):1564–79. doi:10.1016/j.cell.2023.03.003; 37059065 PMC10511214

[55] Cabral-Pacheco GA, Garza-Veloz I, Castruita-De la Rosa C, Ramirez-Acuña JM, Perez-Romero BA, Guerrero-Rodriguez JF, et al. The roles of matrix metalloproteinases and their inhibitors in human diseases. Int J Mol Sci. 2020;21(24):9739. doi:10.3390/ijms21249739; 33419373 PMC7767220

[56] Chu X, Tian W, Ning J, Xiao G, Zhou Y, Wang Z, et al. Cancer stem cells: advances in knowledge and implications for cancer therapy. Sig Transduct Target Ther. 2024;9(1):170. doi:10.1038/s41392-024-01851-y; 38965243 PMC11224386

[57] Clark DW, Palle K. Aldehyde dehydrogenases in cancer stem cells: potential as therapeutic targets. Ann Transl Med. 2016;4(24):518. doi:10.21037/atm.2016.11.82; 28149880 PMC5233526

[58] Lavudi K, Nuguri SM, Pandey P, Kokkanti RR, Wang QE. ALDH and cancer stem cells: pathways, challenges, and future directions in targeted therapy. Life Sci. 2024;356:123033. doi:10.1016/j.lfs.2024.123033; 39222837

[59] Li Z. CD133: a stem cell biomarker and beyond. Exp Hematol Oncol. 2013;2(1):17. doi:10.1186/2162-3619-2-17; 23815814 PMC3701589

[60] Ubago-Guisado E, Rodríguez-Barranco M, Ching-López A, Petrova D, Molina-Montes E, Amiano P, et al. Evidence update on the relationship between diet and the most common cancers from the European prospective investigation into cancer and nutrition (EPIC) study: a systematic review. Nutrients. 2021;13(10):3582. doi:10.3390/nu13103582; 34684583 PMC8540388

[61] Minhajat R, Harjianti T, Rasyid H, Bukhari A, Chaidir Islam I, Zainal ATF, et al. Colorectal cancer patients’ outcome in correlation with dietary and nutritional status: a systematic review. Ann Med. 2023;55(2):2281662. doi:10.1080/07853890.2023.2281662; 38113874 PMC10986434

[62] Encarnação JC, Abrantes AM, Pires AS, Botelho MF. Revisit dietary fiber on colorectal cancer: butyrate and its role on prevention and treatment. Cancer Metastasis Rev. 2015;34(3):465–78. doi:10.1007/s10555-015-9578-9; 26224132

[63] Karumuru V, Dhasmana A, Mamidi N, Chauhan SC, Yallapu MM. Unveiling the potential of urolithin a in cancer therapy: mechanistic insights to future perspectives of nanomedicine. Nanotheranostics. 2025;9(2):121–43. doi:10.7150/ntno.110966; 40568370 PMC12188533

[64] Al-Kabani A, Huda B, Haddad J, Yousuf M, Bhurka F, Ajaz F, et al. Exploring experimental models of colorectal cancer: a critical appraisal from 2D cell systems to organoids, humanized mouse avatars, organ-on-chip, CRISPR engineering, and AI-driven platforms-challenges and opportunities for translational precision oncology. Cancers. 2025;17(13):2163. doi:10.3390/cancers17132163; 40647462 PMC12248563

[65] Antunes N, Kundu B, Kundu SC, Reis RL, Correlo V. *In vitro* cancer models: a closer look at limitations on translation. Bioengineering. 2022;9(4):166. doi:10.3390/bioengineering9040166; 35447726 PMC9029854

