# Supplementary material for: Unveiling the Anticancer Potential of Urolithin A in Colorectal Cancer: A Systematic Review
Source: Oncol Res. 2026 Jan 19;34(2):3. doi: 10.32604/or.2025.070276 (PMC12868977; doi:10.32604/or.2025.070276)
Supplement: Supplementary file 2 [file OncolRes-34-70276-s002.docx]

**Table S1:** Risk of bias assessment of the included studies (n=15) using the Toxicological data Reliability Assessment Tool (ToxRTool) ^18^ for *in vitro* studies. Each of the eighteen criteria was scored with either “0” (criterion not met) or “1” (criterion met).

| Ref. | Criteria Group | | | | | | | | | | | | | | | | | | | Total | |
| --- | --- | --- | --- | --- | --- | --- | --- | --- | --- | --- | --- | --- | --- | --- | --- | --- | --- | --- | --- | --- | --- |
|  | I: Test substance identification | | | | II: Test system characterization | | | III: Study design description | | | | | | IV: Study results documentation | | | V: Plausibility of study design and data | | |  |  |
|  | Test substance identification | Purity of substance | Source of substance | Properties of substance | Test system description | Source of test system | Properties of test system | Method of administration | Concentration | Exposure duration | Negative controls | Positive controls | Replicates | Study endpoints and methods | Study results | Statistical methods | | Study design | Results reliability | |  |
| ^20^ | 1 | 1 | 1 | 1 | 1 | 1 | 1 | 1 | 1 | 1 | 1 | 0 | 1 | 1 | 0 | 1 | | 1 | 1 | | 16 |
| ^21^ | 1 | 1 | 1 | 1 | 1 | 1 | 1 | 1 | 0 | 1 | 1 | 0 | 1 | 1 | 0 | 1 | | 1 | 1 | | 15 |
| ^22^ | 1 | 1 | 1 | 1 | 1 | 1 | 1 | 1 | 1 | 1 | 1 | 1 | 1 | 1 | 1 | 1 | | 1 | 1 | | 18 |
| ^23^ | 1 | 1 | 1 | 1 | 1 | 1 | 1 | 1 | 1 | 1 | 1 | 0 | 1 | 1 | 1 | 1 | | 1 | 1 | | 17 |
| ^24^ | 1 | 0 | 1 | 1 | 1 | 1 | 1 | 1 | 1 | 1 | 1 | 0 | 1 | 1 | 1 | 1 | | 1 | 1 | | 16 |
| ^25^ | 1 | 1 | 1 | 1 | 1 | 1 | 1 | 1 | 1 | 1 | 1 | 1 | 1 | 1 | 1 | 1 | | 1 | 1 | | 18 |
| ^26^ | 1 | 0 | 0 | 1 | 1 | 0 | 1 | 1 | 0 | 1 | 1 | 0 | 1 | 1 | 0 | 1 | | 1 | 0 | | 11 |
| ^27^ | 1 | 0 | 1 | 1 | 1 | 1 | 1 | 1 | 1 | 1 | 1 | 0 | 1 | 1 | 0 | 1 | | 1 | 1 | | 15 |
| ^28^ | 1 | 1 | 1 | 1 | 1 | 1 | 1 | 1 | 1 | 1 | 1 | 1 | 1 | 1 | 0 | 1 | | 1 | 1 | | 17 |
| ^13^ | 1 | 1 | 1 | 1 | 1 | 1 | 1 | 1 | 1 | 1 | 1 | 0 | 1 | 1 | 0 | 1 | | 1 | 1 | | 16 |
| ^29^ | 1 | 1 | 1 | 1 | 1 | 1 | 1 | 1 | 1 | 1 | 1 | 0 | 1 | 1 | 0 | 1 | | 1 | 1 | | 16 |
| ^30^ | 1 | 0 | 0 | 1 | 1 | 1 | 1 | 1 | 1 | 1 | 1 | 1 | 1 | 1 | 0 | 1 | | 1 | 1 | | 15 |
| ^12^ | 1 | 1 | 1 | 1 | 1 | 1 | 1 | 1 | 1 | 1 | 1 | 0 | 0 | 1 | 0 | 1 | | 1 | 1 | | 15 |
| ^15^ | 1 | 0 | 1 | 1 | 1 | 1 | 1 | 1 | 1 | 1 | 1 | 0 | 1 | 1 | 0 | 1 | | 1 | 1 | | 15 |
| ^32^ | 1 | 0 | 1 | 1 | 1 | 1 | 1 | 1 | 0 | 1 | 1 | 1 | 0 | 1 | 0 | 1 | | 1 | 1 | | 14 |

Note: Ref, reference.
